# N-terminal intrinsic disorder is an ancestral feature of Gγ subunits that influences the balance between different Gβγ signaling axes in yeast

**DOI:** 10.1016/j.jbc.2023.104947

**Published:** 2023-06-22

**Authors:** Xinya Su, Yui Tik Pang, Wei Li, J.C. Gumbart, Joshua Kelley, Matthew Torres

**Affiliations:** 1 School of Biological Sciences, Georgia Institute of Technology, Atlanta, Georgia, USA; 2 School of Physics, Georgia Institute of Technology, Atlanta, Georgia, USA; 3 Southeast Center for Mathematics and Biology, Georgia Institute of Technology, Atlanta, Georgia, USA; 4 Department of Molecular and Biomedical Sciences, University of Maine, Orono, Maine, USA

**Keywords:** intrinsically disordered region, heterotrimeric G protein, biased signaling, protein stability, G gamma subunit, G protein signaling

## Abstract

Activated G protein-coupled receptors promote the dissociation of heterotrimeric G proteins into Gα and Gβγ subunits that bind to effector proteins to drive intracellular signaling responses. In yeast, Gβγ subunits coordinate the simultaneous activation of multiple signaling axes in response to mating pheromones, including MAP kinase (MAPK)-dependent transcription, cell polarization, and cell cycle arrest responses. The Gγ subunit in this complex contains an N-terminal intrinsically disordered region that governs Gβγ-dependent signal transduction in yeast and mammals. Here, we demonstrate that N-terminal intrinsic disorder is likely an ancestral feature that has been conserved across different Gγ subtypes and organisms. To understand the functional contribution of structural disorder in this region, we introduced precise point mutations that produce a stepwise disorder-to-order transition in the N-terminal tail of the canonical yeast Gγ subunit, Ste18. Mutant tail structures were confirmed using circular dichroism and molecular dynamics and then substituted for the wildtype gene in yeast. We find that increasing the number of helix-stabilizing mutations, but not isometric mutation controls, has a negative and proteasome-independent effect on Ste18 protein levels as well as a differential effect on pheromone-induced levels of active MAPK/Fus3, but not MAPK/Kss1. When expressed at wildtype levels, we further show that mutants with an alpha-helical N terminus exhibit a counterintuitive shift in Gβγ signaling that reduces active MAPK/Fus3 levels whilst increasing cell polarization and cell cycle arrest. These data reveal a role for Gγ subunit intrinsically disordered regions in governing the balance between multiple Gβγ signaling axes.

Most hormones and neurotransmitters bind to and activate transmembrane G protein-coupled receptors (GPCRs), which in turn activate heterotrimeric G proteins (Gαβγ) that transduce the signal intracellularly (1). This is achieved when ligand/receptor binding catalyzes nucleotide exchange on the Gα subunit, which promotes dissociation of the heterotrimer into Gα and a Gβγ dimer, each of which binds to a distinct subset of effector proteins that transmit the signal throughout the cell. Signaling proceeds until the Gα subunit hydrolyzes GTP back to GDP, a process accelerated by regulators of G protein signaling proteins that promote signal inactivation and reassociation of the heterotrimeric complex (2, 3, 4).

G protein signaling systems are highly conserved among eukaryotes, including the budding yeast *Saccharomyces cerevisiae*, wherein a single canonical heterotrimeric G protein signaling pathway controls the process of yeast mating that is initiated by the binding of α-factor mating pheromones to the pheromone GPCR, Ste2 (5). Activation of the receptor triggers nucleotide exchange on yeast Gα (Gpa1) and the release of yeast Gβγ (Ste4/18). Once free, multiple Ste4/18 molecules nucleate the assembly of what’s been referred to as a “metastable factory” of multiple protein complexes facilitated by three distinct Gβγ effectors (Ste5, Far1, and Ste20) that drive different signaling axes in the cell (6, 7, 8, 9, 10). Ste5 is a MAP kinase (MAPK) scaffold protein that controls a MAPK/transcriptional signaling axis and promotes a phosphorylation cascade resulting in the activation of ERK-like MAPKs Fus3 and Kss1, which drive a transcriptional mating response. Far1 serves a multifunctional role as a scaffold for Bem1 and Cdc24 that coordinate a morphological/cell polarization response and as an inhibitor of cyclin-dependent kinase activity that promotes cell cycle arrest in response to pheromone. Ste20 is a p21-activated protein kinase that promotes activation of both the Ste5 and Far1 signaling axes by serving as the initiator kinase for the Ste5-dependent MAPK cascade and as a direct target of Cdc42, a Rho GTPase that is activated by the Far1/Bem1/Cdc24 signaling axis. Under typical conditions in wildtype cells, the Ste5 axis is the dominant signaling response since Fus3 is required for processes upstream of the Far1 axis (11). Consequently, pheromone activation of the Ste5 axis and Fus3 can occur in the absence of Far1, but cell polarization and cell cycle arrest mediated by the Far1 axis do not typically occur without Fus3 (12). This relationship is not completely fixed, however, as overexpression of components in the Far1 signaling axis (*i.e.*, Far1 or Bem1) can bias cells toward the polarization and cell cycle arrest responses independent of Fus3 (8, 13).

In typical models of Gβγ signaling, the Gγ subunit is often described as a membrane anchor for the Gβ subunit, a function enabled by prenylation of its C-terminal CAAX box (14, 15, 16, 17). However, emerging evidence from mammals (18, 19, 20, 21) and more recently from yeast (22, 23) show that Gγ subunits are also governors of Gβγ signaling. For example, in mammals, longstanding evidence suggests that in response to receptor activation, Gγ controls the kinetics of Gβγ translocation to intracellular membranes by tuning membrane dissociation rate (24). Similarly, in humans, which express 12 different Gγ and five different Gβ subunits, the identity of the Gγ subunit defines the localization and signaling responses of different Gβ/Gγ pairs (25). Activation of some mammalian Gβγ effectors, such as PI3Kγ and PLCβ, has also been shown to be Gγ-subtype-specific (26, 27).

A growing body of evidence suggests that the N termini of Gγ subunits play an important role in their governance of Gβγ signaling (22, 23, 28). This is somewhat surprising due to their relatively short length and characteristic intrinsic structural disorder, which makes them invisible in X-ray crystallography structures. Despite this, the existence of intrinsically disordered N termini is a universal feature among reviewed Gγ subunit sequences, suggesting the possibility that they have been retained through natural selection (28). Intrinsically disordered regions (IDRs), polypeptide segments that do not adopt stable secondary or tertiary structures in otherwise canonically folded proteins (29), represent 40 to 50% of the proteomic sequences in eukaryotes and up to 80% in viruses (30, 31). A growing body of evidence suggests that they provide an array of functional advantages to proteins in which they are found, such as scaffolding, conformational flexibility, and regulation by posttranslational modification, to name a few (32, 33, 34, 35, 36). Such advantages allow IDRs to play diverse roles in cell signaling and regulation processes where multiple proteins are involved and quick or highly regulated transitions from active to inactive states are required. Classic examples include the C-terminal tails of GPCRs, which serve as phosphorylation-rich arrestin scaffolds that drive G protein–independent GPCR signaling (37); the N-terminal tails of histones that serve as epigenetic landmarks for chromatin remodeling complexes (38); or the C-terminal tails of RNA polymerase II enzymes that govern gene transcription initiation and elongation (39). Given the universality of N-terminal intrinsic disorder across all members of the Gγ subunit family, we sought to investigate its evolutionary origins and determine the degree of its functional importance for a canonical G protein γ subunit, yeast Ste18.

## Results

### N-terminal intrinsic disorder is an ancestral trait retained to varying degrees across the Gγ protein family

**Figure 1:**
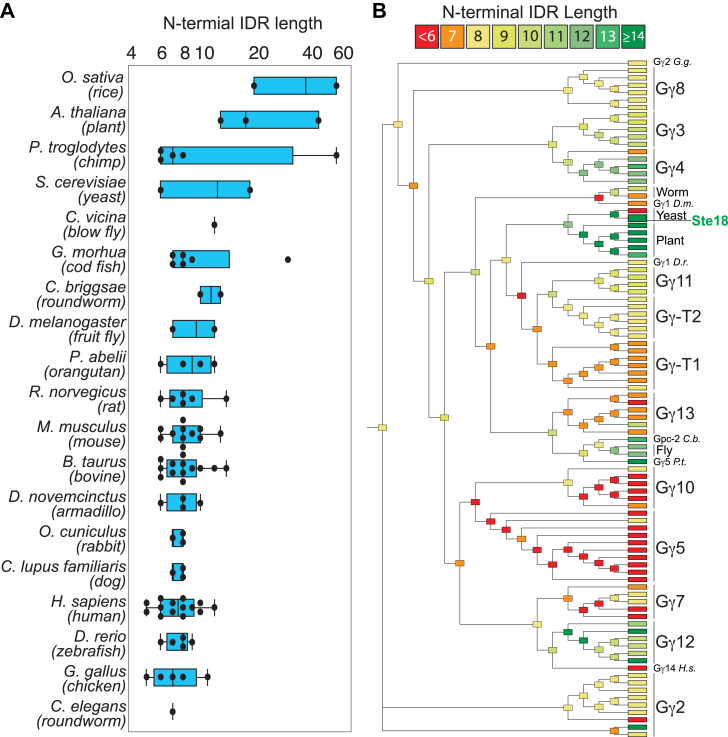
**N-terminal intrinsic disorder is an ancestral feature of the Gγ subunit family.***A*, distribution of predicted disorder lengths of Gγ N-terminal regions from commonly studied organisms. Disordered regions were predicted by IUPred2A. Data shown on log scale. *B*, phylogenetic tree based on ancestral sequence reconstruction of Gγ sequences in (*A*) and color-coded by the predicted length of N-terminal disorder. Fully labeled version found in Table S1-c. IDR, intrinsically disordered region.

N-terminal intrinsic disorder is a structurally conserved feature of modern-day Gγ subunits, suggesting that it has been retained throughout evolution and is therefore more likely to be important for Gγ function. On average, the disordered lengths of Gγ N termini ranges from 5 to 14 amino acids across commonly studied organisms but can be found at longer lengths in plant, chimp, yeast, and fish (Fig. 1*A* and Table S1). To determine whether intrinsic disorder length was a new or ancestral feature of Gγ subunits, we approximated the molecular evolution of this disordered region using ancestral sequence reconstruction based on these and other well studied organisms. We then quantified the length of N-terminal disorder for each node in the reconstructed phylogenetic tree. The resulting analysis suggests a universal common ancestral sequence with an eight amino acid-long disordered N-terminal tail that has been retained or undergone contraction to varying degrees for most subclasses (*e.g.*, Gγ2, Gγ5, Gγ7, Gγ8, Gγ10, Gγ13, Gγ-T1/T2) but has also expanded in other subclasses (*e.g.*, Gγ3, Gγ4, Gγ11, Gγ12 as well as plant and fly Gγ subunits) (Fig. 1*B*).

### A genetic approach to evaluate the importance of tail structure on Gγ function

**Figure 2:**
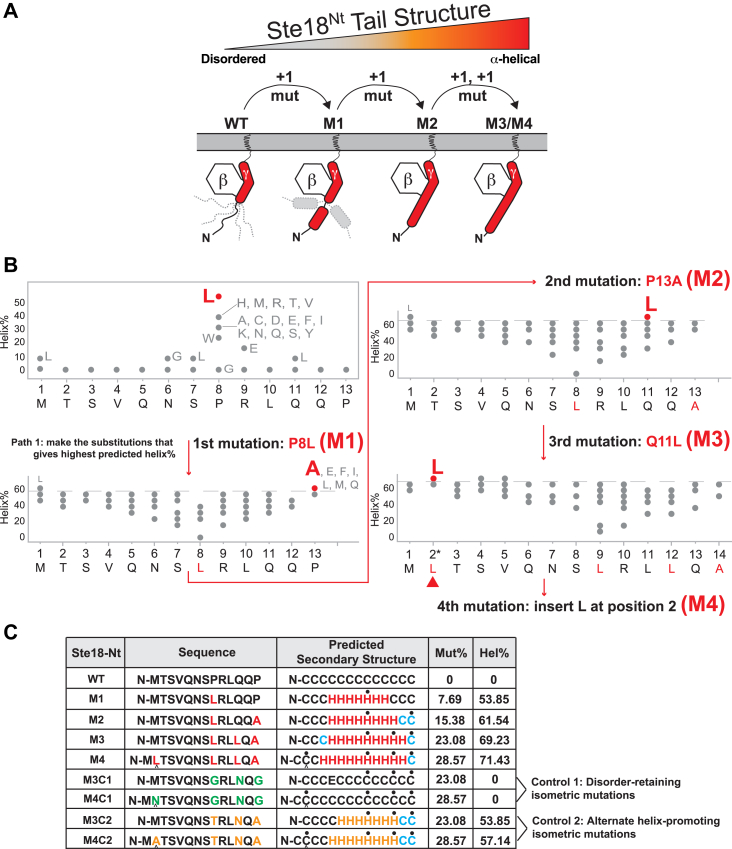
**Identification of a minimal set of mutations that convert the Ste18-Nt from a disordered to an α-helical structure.***A*, schematic summary of the strategy to change Ste18-Nt structure by introducing amino acid substitutions to the tail. *B*, iterated amino acid substitution path to achieve highest predicted helix% in Ste18^Nt^. *C*, table of helix-stabilizing and isometric control mutations incorporated into the Ste18 N-terminal IDR. In the sequence column, helix-stabilizing mutations are shown in *red*. C1 mutations intended to retain the intrinsic disordered state are shown in *green*. C2 mutations intended to provide an alternate helix-stabilizing mutation set are shown in *orange*. Predicted secondary structures are based on PEP2D predictions for the peptide in isolation (*black*, *red*, *orange* colors) or as an extension of the full-length Ste18 protein (where C = random coil and H = alpha helix). Residue positions that are predicted as random coil in the peptide but predicted as alpha-helix in the full-length protein are shown in *blue*. *Black dots* track the point mutation positions in the peptide with respect to the secondary structure prediction. *Black triangles* track the insertion position in M4. The mutation percentage relative to the peptide sequence length (Mut%) and the predicted alpha-helical percentage (Hel%) are shown. Control substitutions including disorder-preserving and alternative helix-promoting mutants are indicated on the *right*. IDR, intrinsically disordered region.

Considering that intrinsic disorder is a structurally conserved feature of Gγ subunits, we asked whether its contribution to Gγ function could be determined. We concluded that inducing a gradual transition from a fully disordered to a fully ordered (helical) state, whereby each transition is characterized structurally *in vitro* and functionally *in vivo*, would be the best approach that also avoids chemical means of structural alteration that are unnatural. To test this approach, we devised an iterative strategy in which a minimal number of single amino acid substitutions could be identified and introduced into the yeast Gγ subunit, Ste18, to gradually produce a fully helical N terminus that is contiguous with the naturally occurring alpha helix structure in the body of the protein (Fig. 2*A*). In each iteration, we analyzed the secondary structure of the N-terminal region using the *PEP2D* algorithm, followed by selection of the N-terminal position and point substitution that would produce the greatest increase in helical structure (Fig. 2*B*) (see Experimental procedures). This mutant was then carried through subsequent iterations to identify the next best mutation until a plateau was reached beyond which increased helical content was not observed. This process produced four distinct Ste18 N-terminal (Ste18^Nt^) mutants (M1–M4), the last of which (M4) included the insertion of an amino acid at position two that was necessary to push the helical content beyond what was possible with substitution alone (Fig. 2*C*). We further made two sets of control mutations: Control 1 (C1) in which each mutation position in M3 or M4 was instead substituted with amino acids that preserve disordered structure and Control 2 (C2) in which we substituted residues at the same positions using amino acids with wildtype-like physicochemical properties predicted to stabilize alpha helical structure (Figs. 2*C* and S1).

### Predicted amino acid substitutions establish a gradient of Ste18 N-terminal structures ranging from fully disordered to alpha helix *in vitro*

**Figure 3:**
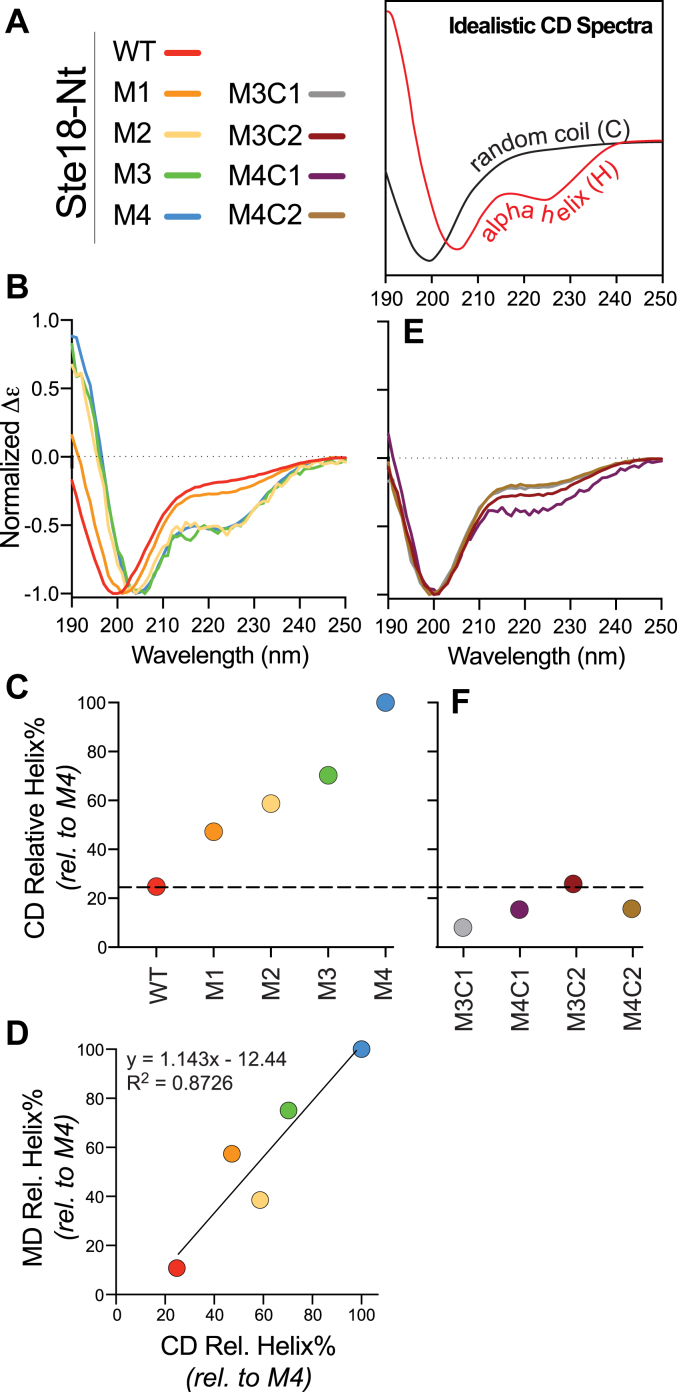
**Validation of Ste18^Nt^Nt structural changes induced by amino acid substitution.***A*, model examples of standard random coil and α-helix CD spectra. *B*, CD spectra of synthetic WT and helix-stabilizing mutant peptides. *C*, quantification of relative helix percentage in WT and M1-M4 peptides based on CD spectra. *D*, correlation between the relative helix percentages of WT and M1-M4 peptides determined by CD and MD. MD data represent the average across six replicate simulations. *E*, CD spectra of synthetic control peptides. *F*, quantification of relative helix percentage for synthetic control peptides based on CD spectra. CD, circular dichroism; MD, molecular dynamics.

Having established a set of mutants predicted to stabilize an increasing degree of alpha helical structure in the Ste18 N terminus, we synthesized tail sequences as peptides and analyzed each using circular dichroism (CD) spectroscopy. CD produces readily distinguishable spectra that can reveal the secondary structure [disordered/random coil (C), alpha-helix (H), or extended beta-sheet (E)] of proteins and peptides. The shape and amplitude of a spectrum can also provide a quantitative measure of secondary structure content (40, 41). Whereas disordered structures have a negative band around 200 nm, α-helixes produce a positive band near 193 nm and negative bands near 208 nm and 222 nm (Fig. 3*A*). As expected, wildtype Ste18^Nt^ produced a CD spectrum characteristic of intrinsically disordered random coils (Fig. 3*B*). M1 showed a slight deviation from wildtype with a noticeable trough at 193 nm that is expected for alpha helical proteins and that was gradually accentuated in M2 and to a greater extent in M3 and M4. Algorithmic extraction of the helical content from each CD spectrum quantitatively demonstrated that a gradual increase in helical content across each proteoform had been achieved with the chosen set of mutations, which was further corroborated by molecular dynamics (MD) simulation of each peptide sequence (Figs. 3, *C* and *D* and S2).

Control peptides harboring isometric mutations intended to retain an intrinsic disordered tail structure with three or four mutations (M3C1, M4C1), also behaved as expected and produced spectra characteristic of random coils (Fig. 3, *E* and *F*). However, unexpectedly, C2 control peptides remained disordered despite being predicted to have ∼54 to 57% alpha-helical propensity (Fig. 2*C*). Taken together, these data demonstrate the ability to encode a gradual transition in Ste18^Nt^ from structurally disordered to fully alpha-helical within as few as four stepwise mutations. Moreover, these data show that genetically encoded tail structures are sequence specific and that not all substitutions predicted to stabilize a particular structural state will do so.

### Ste18 abundance is sensitive specifically to N-terminal helix-stabilizing mutations

**Figure 4:**
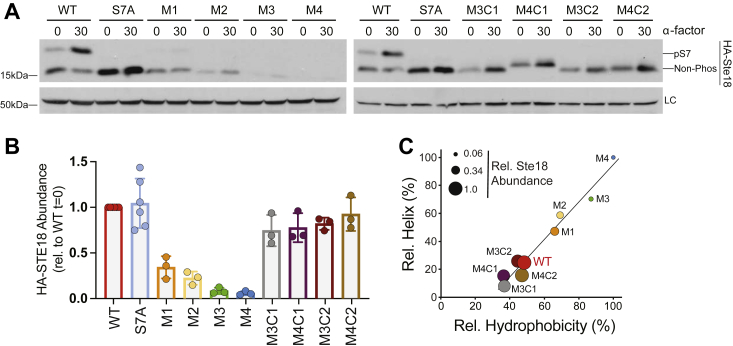
**Helix-stabilizing N-terminal mutations promote a decrease in steady-state level of Ste18.** WT or the indicated mutant yeast strains were treated with 3 μM α-factor (α-F) for 0 or 30 min before being subjected to SDS-PAGE and immunoblotting analysis with anti-HA antibody. *A*, representative immunoblots for WT, S7A, helix-stabilizing mutant (M1, M2, M3, M4), and control mutant (M3C1, M4C1, M3C2, M4C2) cells in response to exposure to 3 μM α-factor. *B*, quantification of relative HA-Ste18 abundance from experiments represented in (*A*). Experiments were performed with three biological replicates. *C*, three-way correlation analysis of measured relative helix% (relative to M4), measured relative Ste18 abundance (indicated by point size relative to WT) and calculated relative hydrophobicity% (relative to M4) in Ste18 isoforms. Quantitative data are based on three biological replicates. LC, loading control (yeast G6PDH); pS7, Ste18 phosphorylation at Ser7; Non-Phos, nonphosphorylated Ste18.

To evaluate the role of N-terminal intrinsic disorder on the function of Gγ/Ste18 *in vivo*, we replaced the endogenous yeast *STE18* gene with helix-stabilizing mutants M1–M4 or with isometric mutation control sequences C1 or C2. We made two versions of each strain, one in which the gene was replaced with nothing more than the N-terminal mutations and another in which each also contained an HA epitope tag for immunoblot detection. As an additional control, we included Ste18^S7A^, which prevents negative-feedback phosphorylation of Ste18^Nt^ in response to pheromone stimulation. This was important since each mutant in this study contained a mutation to proline 8 that is required for negative feedback MAPK phosphorylation of serine 7 (22). Protein steady-state levels were measured for each strain under unstimulated or pheromone-stimulated conditions, revealing a negative correlation between the number of helix-stabilizing mutations and Ste18 abundance relative to wildtype cells (Fig. 4, *A* and *B*). This effect was not observed in any of the control cells harboring amino acid substitutions at the same positions that retain a disordered state suggesting that helix-stabilizing mutations, but not any mutation, cause a change in abundance of the subunit. Further investigation revealed a close, three-way correlation between the measured helix percentage, measured abundance, and calculated hydrophobicity of each Ste18 tail (Fig. 4*C*). Thus, despite prediction, not all mutations are capable of stabilizing an alpha-helical N-terminal tail structure in Ste18. Moreover, mutations M1-M4 simultaneously increase the relative amount of alpha helical and hydrophobic character of the tail. As such, further reference to “helix-stabilizing mutations” refers to both of these inseparable characteristics.

### The effect of helix-stabilizing mutations on Ste18 abundance is proteasome-independent

The decrease in Ste18 steady-state levels observed for M2–M4 was not dependent on ubiquitin-mediated proteolysis since proteasomal inhibition did not lead to specific accumulation of Ste18 or Ste18-Ub proteoforms for the M4 mutant (Fig. S3). We also could not find evidence that these mutations resulted in abnormal aggregation of Ste18, as has been recently reported under conditions of extraordinarily high cell density (42). Surprisingly, comparative analysis of predicted mRNA secondary structure does suggest the potential for unique structures to form for M2–M4 that are adjacent to the translation start site, which we speculate could disrupt translation initiation of these mRNAs (Fig. S4). Consistent with this hypothesis, we did not observe similar disruptions in the predicted mRNA structure for any of the control sequences despite their mutation at the same positions as M3 and M4. Thus, helix-stabilizing N-terminal mutations introduced using species-specific optimal codons promotes a decrease in steady-state levels of Ste18 by an unknown mechanism that is not affected by isometric mutations that retain the naturally intrinsic disordered state.

### N-terminal helix-stabilizing mutations dampen the Ste5 signaling axis while promoting the Far1 signaling axis

**Figure 5:**
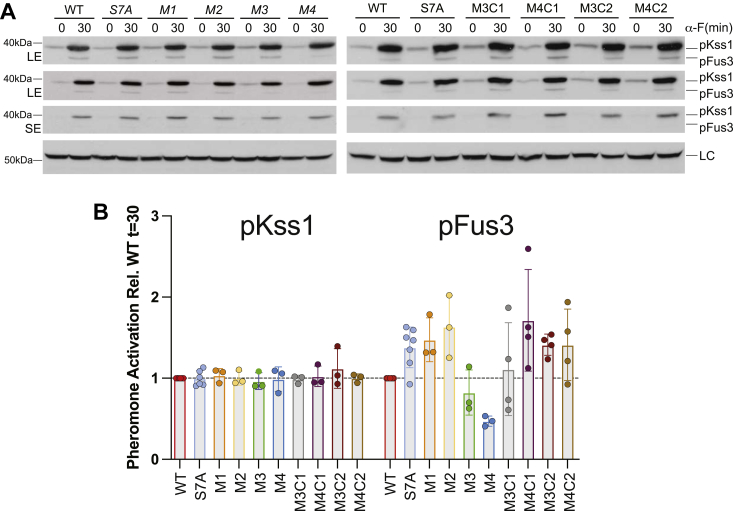
**Effect of underexpressed Ste18-Nt helix-stabilizing mutants on the abundance of pheromone-activated Kss1 and Fus3.** WT or the indicated mutant yeast strains were treated with 3 μM α-factor for 0 or 30 min before being subjected to SDS-PAGE and immunoblotting analysis with phospho-p44/42 MAPK antibody. *A*, representative phospho-MAPK immunoblots in WT, S7A, helix-stabilizing mutant (M1, M2, M3, M4), and control mutant (M3C1, M4C1, M3C2, M4C2) cells in response to 3 μM α-factor. *B*, quantified abundance of activated Kss1 or Fus3 in the mutant *versus* wildtype cells (mutant/wt) on the same immunoblot and based on three to four biological replicates. LC, loading control (yeast G6PDH); LE, long exposure; pFus3, activated Fus3; pKss1, activated Kss1; SE, short exposure.

Considering the established role of free Ste4/18 in coordination of a Ste5 and a Far1 signaling axis, we next tested the axis-specific functional effects of Ste18 helix-stabilizing mutations. In this case, strains lacking the N-terminal HA tag were utilized so as to recapitulate the natural N terminus of the protein and within these strains we measured the level of pheromone-dependent activation for MAPKs Fus3 and Kss1. We first tested the effect of each mutant and control being expressed from the *STE18* promoter at the endogenous genomic locus. Basal activation of Fus3 was undetectable in these strains (Fig. 5*A*). Since any uncontrolled dissociation of Gα and Gβγ leads to pheromone-independent (*i.e.*, basal) MAPK activation (43), this suggests that helix-stabilizing mutations do not disrupt the stability of the Gαβγ heterotrimer. Under stimulated conditions, we found that signaling was still possible for each mutant even despite the dramatic difference in Ste18 abundance across each mutant (see Fig. 4), indicating that G protein activation was also unperturbed (Fig. 5*B*). None of the mutations altered the pheromone response of active Kss1 compared to wildtype cells. However, Fus3 was distinctively affected. Mutants M1 and M2, which are expressed at ∼25% of wildtype Ste18 exhibited slightly elevated (∼1.5×) levels of active Fus3 that were comparable to the S7A control strain that cannot undergo negative feedback phosphorylation. However, the addition of two more helix-stabilizing mutations beyond this in M3 and M4 significantly dampened the level of active Fus3, while isometric M3 and M4 controls (both C1 and C2) matched the response of the S7A control (Fig. 5).

**Figure 6:**
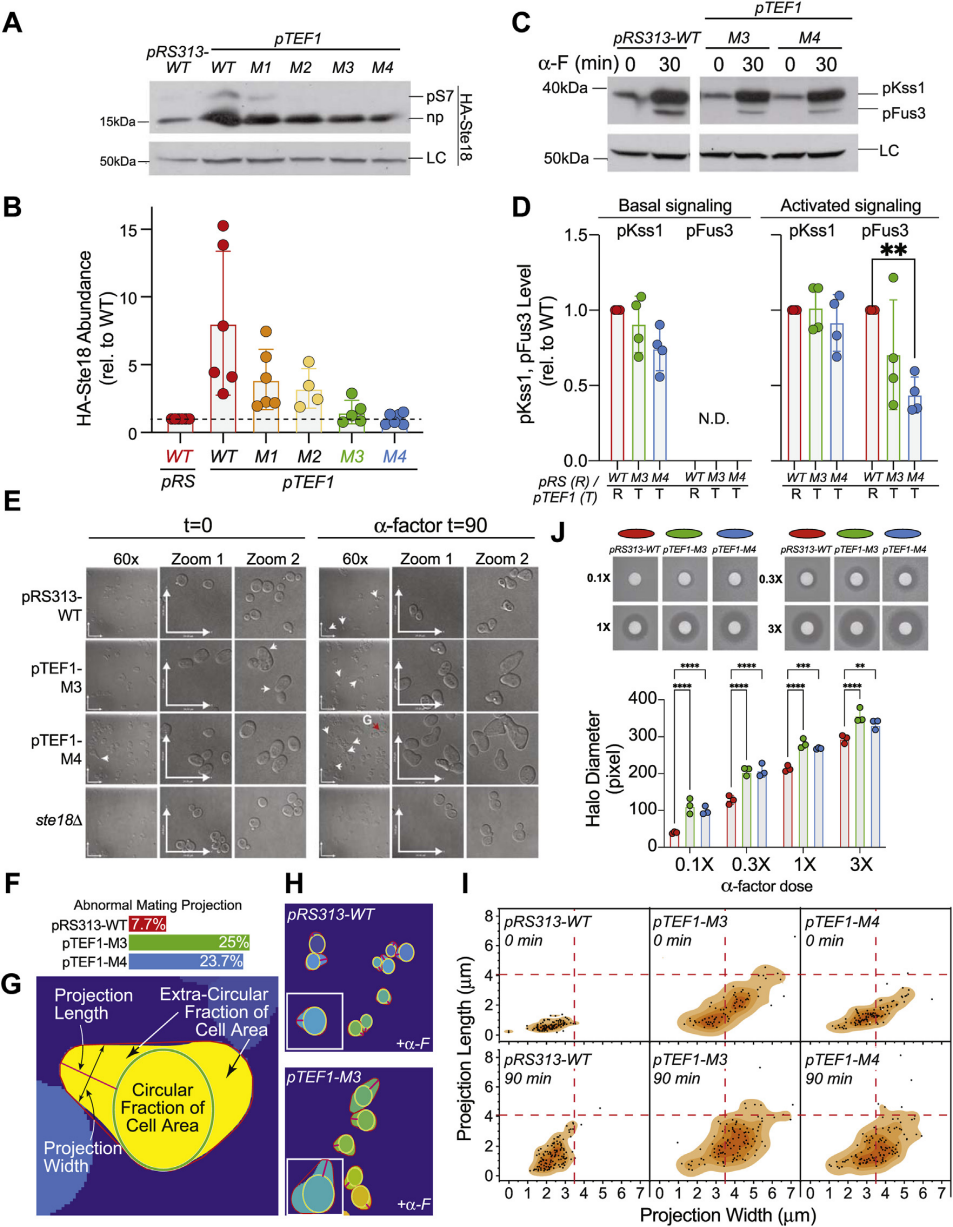
**Differential effect of comparably expressed Ste18^Nt^- helix-stabilizing mutants on pheromone-dependent MAPK activation, cell polarization, and cell cycle arrest.***A*, representative anti-HA immunoblot showing differential abundance of HA-Ste18 expressed in *ste18Δ* cells transformed with *pRS313-HASTE18* centromeric plasmid or *pTEF1-HASTE18-M1/M2/M3/M4* overexpression plasmid. *B*, quantification of HA-Ste18 abundance relative to HA-Ste18-WT from experiments represented in (*A*). *C*, representative immunoblot of phospho-MAPK before and after 30-min treatment with 3 μM α-factor. *ste18Δ* cells transformed with *pRS313-STE18* or *pTEF1-STE18-M3/M4* were treated with 3 μM α-factor followed by immunoblot analysis with anti-phospho-p44/42 MAPK antibody. *D*, quantification of basal and pheromone-activated MAPKs level from experiments represented in (*C*). *E*, DIC microscopy images of *ste18*Δ yeast harboring *pRS313-WT* or *pTEF1-M3* or *M4* expression plasmids before and after treatment with 3 μM α-factor for 1.5 h. Images are representative of multiple images taken. Scale bars are 24 μm × 24 μm. *F*, manual count of abnormal mating projections produced by each of the indicated plasmid-harboring yeast cells. *G*, schematic diagram of automated yeast mating projection analysis features. DIC image from which this mask was generated is shown in panel *E*. *H*, example autogenerated mask layers used for systematic mating projection analysis. Shown are examples from *pRS313-WT* and *pTEF1-M3* cells in the presence of α-factor. *I*, mating projection length *versus* width plots in the absence (*top*) or presence (*bottom*) of pheromone. *Dashed lines* indicate plot midpoint for the universal scale. *J*, representative image and quantification of halo assay for pheromone-induced cell cycle arrest. Sterile discs were saturated with 3X (3 mM), 1X, 0.3X, or 0.1X α-factor. Experiments were repeated at least three times. Statistical significance was determined for MAPK and HALO results by two-way ANOVA and corrected for multiple comparisons using Tukey’s test. Statistical significance for microscopy results is reported in supplemental information. ∗∗*p* < 0.0021, ∗∗∗*p* < 0.0002, ∗∗∗∗*p* < 0.0001. DIC, differential interference contrast; LC, loading control (yeast G6PDH); ND, not detected; np, nonphosphorylated Ste18; pFus3, activated Fus3; pKss1, activated Kss1; pS7, Ste18 phosphorylated at Ser7.

Since M3 and M4 express at ∼10% of wildtype Ste18 levels, we next sought to determine if a dampened Fus3 response was simply due to a deficiency in M3 and M4 abundance or due to intrinsic qualities of the helix-stabilizing mutations in these cells. Suspecting that we could overcome the differential expression of M1–M4 *via* overexpression, we exogenously overexpressed each mutant and wildtype *STE18* using a constitutively active *pTEF1* promoter (44). In this case, *pTEF1-WT* cells produced between 3- and 15-fold more protein compared to a control plasmid expressing *STE18* from its native promoter, confirming overexpression of the gene (Fig. 6, *A* and *B*). Despite overexpression, we found that the relative expression differences between *pTEF1-ste18* mutants (M1–M4) remained unchanged in comparison to expression that was observed previously from the native promoter (compare with Fig. 4). Overexpression of wildtype *STE18* at this level had a drastic negative effect on downstream MAPK activation, which necessitated that only similarly expressed forms of Ste18 should be functionally compared (Fig. S5). Fortuitously, overexpression using *pTEF1-M3* and *pTEF1-M4* resulted in nearly identical Ste18 levels compared to *pRS313-WT* control, which enabled a direct functional comparison of these three forms of Ste18.

Similar to our observation in cells with endogenously expressed Ste18, we found that the levels of active Kss1 were not significantly affected under either basal or pheromone-activated conditions in cells expressing wildtype-comparable levels of M3 and M4 from the *pTEF1* vector (Fig. 6, *C* and *D*). Active Fus3 was also undetectable for both *pTEF1-M3* and *pTEF1-M4* under basal conditions, again confirming that typical heterotrimeric G protein complexation between Gα and Gβγ is not prominently disrupted by helix-stabilizing mutations as measured by downstream signaling responses. Under pheromone activated conditions, we again confirmed a significant reduction (∼50%) in the levels of active Fus3 for both M3 and M4, confirming that helix-stabilizing mutations but not Ste18 abundance were responsible for the reduced levels of active Fus3 relative to wildtype.

We next evaluated the response from the Far1 signaling axis responsible for driving cell polarization and arrest of the cell cycle in response to pheromone. Since we observed a clear reduction of active Fus3 in M3 and M4 cells, we expected to find anywhere from a dampened to an undetectable effect on cell polarization and arrest. Instead, we found that cells expressing either M3 or M4 were hyperpolarized with abnormally large and elongated mating projections observed at frequencies ∼17% greater than wildtype cells treated with pheromone (Fig. 6, *E* and *F*). Moreover, we also found that untreated *pTEF1-M3/M4* cells exhibited more ovular/slightly elongated shape compared to the very circular shape of *pRS313-WT* cells.

In order to systematically quantify the morphological effects of M3 and M4, we employed the use of a customized image analysis algorithm that automatically quantifies cell morphology (length, width, and circular area) relative to a centered and area-maximized circle (Fig. 6*G*). Typically, wildtype yeast cells are highly circular in shape, before and even after isotropic exposure to pheromone. However, pheromone also promotes cell polarization in the form of a mating projection (aka. Shmoo) that is a generally short, relatively narrow extension away from the centroid circular shape (Fig. 6*H*). In contrast, highly polarized cells deviate from this circular shape, as exemplified by *pTEF-M3/M4 cells*. We quantified this shape change across multiple microscope images of yeast in the absence or presence of pheromone. As expected, *pRS313-WT* cells are significantly more circular as compared to *pTEF1-M3/M4* cells in either the absence or the presence of pheromone, as judged by the fraction of cellular area contained within an area-maximized circle (Fig. S6*A* and Table S2). In the presence of pheromone, the circular area of *pRS313-WT* cells decreases with a concomitant increase in projection length (Figs. 6*I* and S6, *B* and *C*). *pTEF1-M3/M4* cells undergo a similar increase in projection length after pheromone treatment. However, these cells exhibit significantly different and broader distributions across all dimensions (projection length, width, and circular fraction) compared to WT cells (Table S2). Moreover, *pTEF1-M3/M4* projections measured in the absence of pheromone were just as long and wide in range before and after treatment (Fig. 6*I*). Empty vector controls in wildtype *BY4741* cells further demonstrated that this elongated/wider shape is a function of the Ste18 mutants and not due to the vectors themselves (Fig. S7).

Since the Far1 axis is also essential for promoting cell cycle arrest in response to mating pheromone, we also compared each strain for pheromone-induced growth inhibition. We found that *pTEF1-M3/M4* cells undergo significantly greater pheromone-induced cell cycle arrest compared to wildtype cells, which was accentuated at low pheromone dosage and consistent with the hypothesis that N-terminal helix-stabilizing mutations in Ste18 have a positive impact on the Gβγ/Far1 signaling axis (Fig. 6*J*).

Fus3 activation, cell morphology, and cell cycle arrest phenotypes observed for untagged mutants were also recapitulated for HA-tagged forms in the M3 and M4 strains (Fig. S8 and Table S3).

Taken together, these data suggest that helix-stabilizing mutations in the Ste18 N-terminus shift the signaling bias of Gβγ subunits. While the Ste5 signaling axis that governs Fus3 activation and the mating transcriptional response is negatively impacted by helix-stabilizing mutations, the Far1 axis, which controls pheromone-dependent cell polarization and cell cycle arrest, is promoted by such mutations.

## Discussion

Here we have shown that N-terminal intrinsically disordered tails are an evolutionarily conserved and persistent feature of G protein γ subunits. Using a combination of *in vitro*, in silico, and *in vivo* methods, we provide evidence that intrinsic disorder makes an important contribution to Gβγ-dependent G protein signaling and further reveal its contribution to Gβγ signaling bias. Inherent in the study is the caveat that intrinsic disorder and secondary structure in proteins are inevitably convoluted by the unique amino acid propensities and physicochemical properties in either structural type. Therefore, interpretations made from our approach and results must consider both sequence and structure simultaneously.

### Persistence of Gγ-Nt IDRs through evolution suggests that it has broad functional importance in G protein signaling

IDRs are a prominent feature of the proteomes of all living things, from viruses to bacteria to humans. In comparison to prokaryotes in particular, which exhibit an average proteomic intrinsic disorder of ∼20%, eukaryotic IDRs represent between 30 and 50% of proteomic sequences, while virus proteomes range from ∼2% to ∼80% IDR content (31). Thus, one possible conclusion is that IDRs have evolved to expand regulatory complexity of more recently evolved organisms. Using ancestral sequence reconstruction, we provide evidence that Gγ-Nt IDRs are a persistent and diverse structural feature retained to varying degrees in all Gγ subunits. Combined with evidence that the N-terminal IDR impacts the functional output of Gβγ subunits in yeast, the result suggests that these structures provide some selective advantage for G protein signaling systems due to their impact on protein abundance, Gβγ signaling, or both.

### Deconvoluting the contribution of intrinsic disorder to protein function in Gγ subunits

Intrinsically disordered protein termini (N or C) play important roles in the biochemistry of a growing list of proteins such as histones, GPCRs, and RNA polymerases, to name a few. Understanding the contributions of structural disorder, *itself*, is therefore of growing interest (45). Stabilizing a favored protein secondary structure has been done previously in the engineering of peptide biologics in which amino acid substitution and chemical “stapling” by internal isopeptide bonds has been shown to stabilize alpha helical structures of isolated peptide-based drugs (46, 47, 48). However, these approaches are far from ideal for studying IDRs in full-length proteins expressed in living cells since they introduce unnatural, nonproteinaceous features. Here, we established a genetic approach to this challenge, which effectively titrates the amount of helical order contained within the Gγ-Nt IDR (Fig. 2). Our approach is intended to allow the distinction between phenotypes resulting from the gradual shift from disorder to order that provides greater resolution of the effect compared to a single brute force change in structure. To our knowledge, this is the first time such a titration-based genetic approach has been attempted to study an IDR in any system.

The tractability of Gγ N-terminal tails, and especially that of Ste18, couples well with a genetic strategy to gradually transition an IDR to a stable structure, as we have confirmed by CD and MD analysis (Figs. 3 and S2). In the case of Ste18, the effects of helix-stabilizing mutations were less trivial to analyze due to the fact that increasing the number of helix-stabilizing, but not IDR-preserving mutations, causes a decrease in cellular abundance of the protein (Fig. 4). Strikingly, overexpressing the protein up to 15-fold over typical levels has no effect on the relative abundances of helix-stabilizing mutants, which showed the exact same trend in abundance as when expressed normally. Thus, the mechanism by which Ste18 abundance is controlled in response to helix-stabilizing mutations is strong enough to overcome even extreme overexpression levels. We found no evidence to suggest an increase in protein ubiquitination (Fig. S3), nor did we ever observe any evidence of protein aggregation for any of the mutants. However, we did find evidence to suggest that helix-stabilizing mutations, but not control mutations, may alter *STE18* mRNA secondary structure near the translation start site and therefore possibly alter translation efficiency. This mechanism would certainly explain why even overexpression cannot overcome relative differences in abundance across the mutants (Fig. 6, *A* and *B*). More importantly, these results reveal a strong correlation between N-terminal intrinsic disorder and cellular abundance of Ste18. We speculate that similar effects might also be observable for human gamma subunits since their N-terminal tails are generally shorter and mutations in them would be very close to the translation start site. Future work that more deeply explores the relationship between Gγ translation, N-terminal mutations, and mRNA structure could shed light on connections between mRNA structure and protein evolution.

### Effects of Gγ helix-stabilizing mutations on the Gαβγ heterotrimeric complex as gleaned from pheromone-dependent MAPK activation

In yeast, the activation of Fus3 in response to mating pheromone requires nucleotide exchange on Gα and the release of Gβγ. Conversely, disruptions that prevent proper complexation between Gα and Gβγ in the absence of pheromone (*i.e.*, basal conditions) will result in pheromone-independent Fus3 activation. We find that introducing helix-stabilizing mutations in the N-terminal tail of Ste18 does not result in pheromone-independent basal activation nor does it prevent the pheromone-dependent activation of Fus3 (Fig. 6*D*). Taken together, these data suggest that Gγ-Nt helix-stabilizing mutations do not significantly disrupt heterotrimeric G protein function, but rather take their effect on Gβγ/effector function.

### Gγ N-terminal tails: governors of Gβγ signaling bias?

Signaling bias is an emergent concept spawned from the discovery that GPCRs can elicit both G protein and arrestin-mediated signaling responses in which biased activation of one *versus* the other pathway is controlled in part through structural changes (*e.g.*, phosphorylation) in their long C-terminal IDRs (49, 50, 51). At a fundamental level, the concept of signaling bias is not necessarily restricted to one component of a signaling pathway (*e.g.*, GPCRs). For example, an apt description for signaling bias might also include any signaling protein that participates in two or more distinct yet simultaneous processes where a shift in the strength of activation of one process over another can occur under different circumstances. Given this view, three pieces of evidence from this work support the hypothesis that the structure of Ste18-Nt alters bias between the Far1 and Ste5 signaling axes of Gβγ (Figs. 5 and 6). First, in conditions when helix-stabilized Ste18 mutants M3 and M4 are either underexpressed or comparably expressed relative to wildtype, the levels of active Fus3 produced in response to pheromone are significantly dampened by ∼50%. This likely suggests a defect in the ability of the Ste18-M4 to efficiently coordinate a Ste5-dependent MAPK cascade that terminates in the phosphorylation of Fus3, which is supported by previous results showing that changes in N-terminal tail can disrupt the Gβγ/Ste5 interaction (22). Second, when expressed at levels comparable with WT, these mutants exhibit significantly greater pheromone-dependent cell cycle arrest compared to wildtype cells. This points to an enhancement of the Gβγ/Far1 signaling axis since Far1 itself is responsible for controlling pheromone-dependent cell cycle arrest. Third, M3 and M4 exhibit abnormal polarized growth in response to pheromone. This points to a miscoordination of polarization factors like Cdc24 and Bem1 that are essential for the morphological response to pheromone. Another argument in support of this hypothesis is the fact that the M3 and M4 mutants are able to simultaneously decrease the MAPK response while promoting a cell cycle arrest and hyperpolarization responses. This subtle yet important point is extraordinary given that pheromone-activated Fus3 plays an essential role in all Gβγ signaling axes and is effectively upstream of the cell cycle arrest and polarization responses (10, 11). However, previous reports also demonstrate that this essential role of Fus3 can be bypassed *via* overexpression of proteins in the Far1 signaling axis, including Far1 itself and the Far1 interacting protein, Bem1 (8, 13). Interestingly, the cell cycle arrest response in OE-Far1 and OE-Bem1 cells looks similar to that of Ste18-M3 and M4 cells that we report here (Fig. 6*J*).

The data presented here are supported by prior evidence showing that Gγ N termini modulate Gβγ/effector interactions and signaling both in yeast and in humans (18, 22, 23). Additional evidence shows that intrinsic disorder-to-order transitions (modulated by posttranslational modification) can promote dramatic structural changes in IDRs and influence protein interactions (36, 52). Here, we have shown that Gβγ signaling can be directly influenced in part by structural changes to the Gγ N-terminal IDR alone. Further work aimed at understanding the structural dynamics of the Ste18 N-terminal IDR, and its role in Gβγ/effector interactions could provide new avenues for controlling biased signaling in mammalian G protein signaling systems.

### Limitations of the study

Our study employs a genetic approach to investigate the importance of intrinsic disorder in a G protein signaling system. Inevitably, the structure of a protein is dictated by its sequence, and in a genetic approach, the sequence and structure of mutant proteins is necessarily convoluted. We have attempted to control for this fact by the introduction of isometric mutations that do not promote the target structure and in so doing have been able to see that helix-stabilizing but not IDR-preserving mutations alter Ste18 function. That said, it is not clear whether alternative mutations that may stabilize a helix in Ste18-Nt would produce the exact same results as ours, although we suspect it is unlikely since our computational approach to identifying a minimal set of helix-stabilizing substitutions provided very few paths that deviated from our current set (M1–M4) (Fig. 2). Moreover, attempting to make mutants *via* a next best path were not successful in stabilizing helical structures (Figs. S1 and 3). Given the significant impact of mutations in the Ste18 N-terminal tail, further work to deconvolute the sequence/structure/signaling landscape of Gγ subunits may be warranted in the future.

## Experimental procedures

### Yeast strains and plasmids

A complete list of yeast strains used in the study are shown here (Table S4). Unless specified, all strains used in this study were derived from the strain *BY4741* (*MATa leu2Δ met15Δ his3Δ ura3Δ*). All mutants were constructed by delitto perfetto mutagenesis and verified by polymerase chain reaction amplification and dideoxy sequencing (Eurofins Genomics). To construct overexpression plasmids, *STE18* mutants and *HA-STE18* mutants were amplified from relative mutant strains and were cloned downstream of the TEF1 promoter into *pESC-His3-P*_*TEF1*_*-P*_*ADH1*_ (pTEF1) digested with *BamHI* and *SacII* using sequence and ligation-independent cloning. pTEF1 plasmid was provided by Dr Kuntal Mukherjee.

### Media and growth conditions

Depending on the treatment conditions, yeast strains were grown in either YPD growth medium (yeast extract, peptone, and 2% dextrose media) or synthetic complete (SC) or SC dropout medium with 2% dextrose. Cells were grown at 30 °C to mid-log phase (*A*_600_ = 0.75–0.85) followed by treatment with α-Factor peptide hormone (GenScript) at a final concentration of 3 μM for the indicated time. After treatment, cell growth was stopped by the addition of 5% trichloroacetic acid after which cells were immediately harvested by centrifugation at 4000 rpm in an Allegra X-14R Beckman Coulter centrifuge at 4 °C, washed twice with ice-cold Milli-Q water, and frozen at −80 °C.

### Cell lysis and immunoblotting

Cell pellets were subjected to glass bead lysis in the presence of trichloroacetic acid buffer according to the standardized protocol described previously (53). Protein extract concentrations were measured using a DC protein assay (Bio-Rad), normalized, and separated by SDS–polyacrylamide gel electrophoresis with 12.5% or 16% acrylamide. Primary antibodies and dilutions used for immunoblotting included the following: pKss1 and pFus3 (phospho-p44/42 MAPK, Cell Signaling Technologies, catalog no. 9101; 1:500); hemagglutinin antigen epitope (HA; Cell Signaling Technologies, catalog no. 3724; 1:2000); glucose-6-phosphate dehydrogenase (loading control) (Sigma-Aldrich, catalog no. A9521; 1:50,000); and anti-ubiquitin (Abcam, Cat#: ab19247, 1: 500). In all cases, a horseradish peroxidase–conjugated secondary antibody (goat anti-rabbit) (Bio-Rad, catalog no. 1705046; 1:5000) was used for detection. The signal was detected with an enhanced chemiluminescence reagent (PerkinElmer, catalog no. NEL 104001EA) and developed on autoradiography film. In all cases, several exposures were made, and only those exposures for which all signals were below saturation were used for quantification. Quantification was achieved by high-resolution scanning of appropriate films, followed by image densitometry with ImageJ software to quantify signal intensities (54).

### Proteasome inhibition assay

Cells were grown in SC media supplied with 0.1% proline overnight and then diluted to *A*_600_ = 0.2 in SC media supplied with 0.1% proline and 0.003% SDS and grown for 3 to 4 h. Cells were treated with 100 μM MG132 for 3.5 h to inhibit proteasome function followed by harvesting and SDS-PAGE and immunoblot analysis.

### Halo assay

Halo assays for pheromone-induced cell cycle arrest were carried out with *ste18Δ* cells transformed with *pRS313 or pTEF1* plasmids harboring *STE18* or *HA-STE18*. Equal amounts of cells from overnight culture were spread on SC-His plates and allowed to dry for 30 min before placing discs soaked with various concentrations of α-factor peptide pheromone. The plates were incubated at 30 °C for approximately 1 day before scanning and quantifying halo diameters using ImageJ.

### Morphological response assay

Cells transformed with *pRS313* or *pTEF1* plasmids harboring *STE18* or *HA-STE18* were grown at 30 °C to mid-log phase and collected before and after 1.5 h of 3 μM α-factor stimulation. Cell morphology was captured by differential interference contrast confocal microscopy using a PerkinElmer UltraVIEW spinning disk confocal microscope. After 1.5 h of pheromone stimulation, the number of cells forming a clear mating projection *versus* no projection or the number of cells forming normal *versus* abnormal mating projections was counted across three fields captured using a 60× objective.

### Ancestral sequence reconstruction and intrinsic disorder prediction

Protein sequences used for ancestral sequence reconstruction were initially derived from (55) and retrieved from UniProt and NCBInr databases. An expanded set of sequences were identified by capturing reviewed entries contained within InterPro protein family IPR001770. After removal of obsolete entries and the addition of reviewed plant sequences, the total number of unique proteins was 92. Sequence alignment was performed by MAFFT Version 7 (56). Ancestral sequence reconstructions were calculated by ANCESCON using the marginal reconstruction method (57). Ancestral sequences were reconstructed for all internal nodes using an alignment-based rate factor and an alignment-based equilibrium (background) amino acid frequency vector. Disordered regions of all sequences were predicted using IUPred2A (58).

### PEP2D secondary structure prediction

Ste18 N-terminal tail secondary structure prediction was carried out using the PEP2D *mutant peptides module* (59). Ste18 wildtype N-terminal sequence was first used as input to generate the sequences with all possible single mutations at each position and their relative predicted secondary structure. The single mutated sequence with the highest predicted helix% was used as next input, and the mutant sequences and relative predicted secondary structures were generated iteratively until no increase in predicted helix% with further mutations. We note that PEP2D and IUPred2A predictions may sometimes contain some minor differences in the number of predicted disordered residues.

### Circular dichroism spectroscopy

Commercial synthetic Ste18 N-terminal tail and mutant isoforms were synthesized with C-terminal amidation and enriched to 95% purity (GenScript). Lyophilized peptides were reconstituted in deionized water and further diluted as necessary with 10 mM potassium phosphate buffer (pH 7). Methanol was added at 20% final concentration to stabilize the secondary structure of peptides. Far-ultraviolet (190–250 nm) CD spectroscopy was performed on a Jasco J-815 CD spectrometer equipped with a Peltier temperature control unit. A quartz cuvette with a 1 mm path length was used at 20 °C. Measurements were performed with a 50 nm/min scan rate in 0.2-nm steps with a 1 s response time and a 1 nm bandwidth. Single CD spectra were averaged over 15 scans after buffer baseline correction. Secondary structure predictions based on CD spectra were performed with BeStSel (41).

### Molecular dynamics simulation

MD simulations were performed with NAMD2.13 (60) and Amber16 to 18 (61). The CHARMM36m protein force field (62) was used to describe the peptides, and the TIP3P water model (63) was used to describe the solvent and ions. The initial structure of the Ste18 N terminus was prepared with VMD (64) and its plugin Molefacture. All four mutant isoforms of the peptide were constructed, including M1, M2, M3, and M4. Each peptide was placed in a water box, and ions were added to neutralize the system at a concentration of 150 nM NaCl. The final system size was around 20,300 atoms. Each system was equilibrated at 400 K and a constant volume for 4 ns to randomize the starting conformation and then cooled at 298 K and 1 atm for 2 ns using NAMD. Next, six independent 2-μs production runs were performed for each system at 298 K and constant volume using Amber. The temperature was maintained using Langevin dynamics for all simulations, and the pressure was kept at 1 atm using the Langevin piston method when applied. All simulations were performed using a 2-fs time step and under periodic boundary conditions with a cutoff at 12 Å for short-range electrostatic and Lennard-Jones interactions and a switching function beginning at 10 Å. Long-range electrostatic interactions were calculated by the particle mesh Ewald method. System setup, analysis, and visualization were performed with VMD.

### mRNA secondary structure prediction

mRNA secondary structures of HA-Ste18-WT and mutant isoforms M1–M4 were predicted using the RNAfold web server (65), with minimum free energy and partition function folding algorithms. mRNA sequences from position −32 from the translation start site to the translation stop codon were used for comparative analysis.

### Morphology quantitation

Quantitation of the morphology of the mating projection (shmoo) was carried out in MATLAB with a custom script. Cell masks were created using Cellpose (66), and improperly masked images were removed or corrected prior to analysis. Analysis of cell masks was carried out in MATLAB (MathWorks). The body of the yeast was defined by the largest circle that could be inscribed within the mask. The shmoo was defined by the largest circle that could be inscribed in the remainder of the cell that was not segmented into the body. The length of the shmoo was determined by a line defined by the centroids of the two inscribed circles. The ends of the shmoo-line were its intersection with the edge of the cell mask and its intersection with the cell body defined by the inscribed circle. The width of the shmoo was determined by finding the midpoint of the shmoo-line, and then drawing a line through that point, perpendicular to the shmoo-line. The ends of the width-line were determined by where it intersected with the cell periphery. The shmoo had to be longer than one pixel in order to determine a width. In the case that the width-line intersected the cell periphery at only one point (possible in very short shmoos), the width was set to 1. Because the shmoos are defined by deviations of the cell mask from the largest inscribed circle, even cells that are not undergoing a mating response (such as the 0 pheromone controls) will identify very small projections. Circular fraction was defined as the fraction of the mask contained within the largest inscribed circle.

### Statistical analysis

Statistical analyses of MAPK and HALO assay results were performed using Prism software v9 (GraphPad Software) in which case statistical significance was determined by two-way analysis of variance (ANOVA) corrected for multiple comparisons using Tukey’s test. Statistical analysis of microscopy data was performed using JMP 16 (SAS Inc), in which case statistical significance was measured between *pTEF1-M3/M4 versus pRS313-WT* (control) cells using Dunnett’s method.

## Data availability

All data are presented in the paper and associated supporting information.

## Supporting information

This article contains supporting information.

## Conflict of interest

The authors declare that they have no conflicts of interest with the contents of this article.
