# Supplementary material for: N-terminal intrinsic disorder is an ancestral feature of Gγ subunits that influences the balance between different Gβγ signaling axes in yeast
Source: J Biol Chem. 2023 Jun 22;299(8):104947. doi: 10.1016/j.jbc.2023.104947 (PMC10393545; doi:10.1016/j.jbc.2023.104947)
Supplement: Supporting Figures S1–S8 and Tables S2–S4 [file mmc1.docx]

**SUPPORTING INFORMATION**

**N-terminal intrinsic disorder is an ancestral feature of Gγ subunits that influences the balance between different Gβγ signaling axes in yeast**

Xinya Su^1^, Yui Tik Pang^2^, Wei Li^3^, JC Gumbart^2^, Joshua Kelley^4^, and Matthew Torres^1,3^*

^1^School of Biological Sciences, Georgia Institute of Technology, Atlanta GA, 30332

^2^School of Physics, Georgia Institute of Technology, Atlanta GA, 30332

^3^Southeast Center for Mathematics and Biology, Georgia Institute of Technology, Atlanta GA, 30332

^4^Dept. of Molecular and Biomedical Sciences, University of Maine, Orono, ME 04469

**CONTENTS:**

**Table S1.** ANCESCON ancestral sequence reconstruction raw input and output (*External Excel File*)

**Table S2.** Significance test results for microscopy image data analysis. All p-Values were determined using JMP 16 comparison to control using Dunnet’s method, where *pRS313-WT* serves as the control group. Non-circular fraction corresponds to 1-circular fraction.

**Table S3.** Table of helix-stabilizing and isometric control mutations incorporated into the HA-Ste18 N-terminal IDR.

**Table S4.** List of yeast strains used in this study.

**Fig. S1.** Path to introduce alternative helix-promoting substitutions to Ste18^Nt^.

**Fig. S2.** Molecular dynamics simulation replicates for WT and helix-stabilizing Ste18 mutant peptides M1-M4 (refer to Fig. 3).

**Fig. S3.** The effect of inhibiting the 26s proteasome on the cellular abundance of Ste18 mutant M4.

**Fig. S4.** mRNA structure prediction of HA-tagged *STE18-WT* and mutant isoforms.

**Fig. S5.** Immunoblot and image densitometry analysis for activated MAPK/Fus3 and MAPK/Kss1 in *pRS313-WT* versus *pTEF1-WT* cells 0 and 30 minutes post pheromone stimulation.

**Fig. S6.** Comparison of circular fraction, non-circular fraction and yeast cell projection length quantified from DIC images across *pRS313-WT*, *pTEF1-M3*, and *pTEF1-M4* cells.

**Fig. S7.** DIC microscope images of *ste18Δ* and *WT BY4741* cells harboring either *pRS313* or *pTEF1* empty vector treated with or without pheromone for 90 minutes.

**Fig. S8**. Effect of comparably expressed HA-tagged Ste18-Nt helix-stabilizing mutants on pheromone-dependent MAPK activation, cell polarization, and cell cycle arrest.

**Table S2.** **Significance test results for microscope image analysis data.**


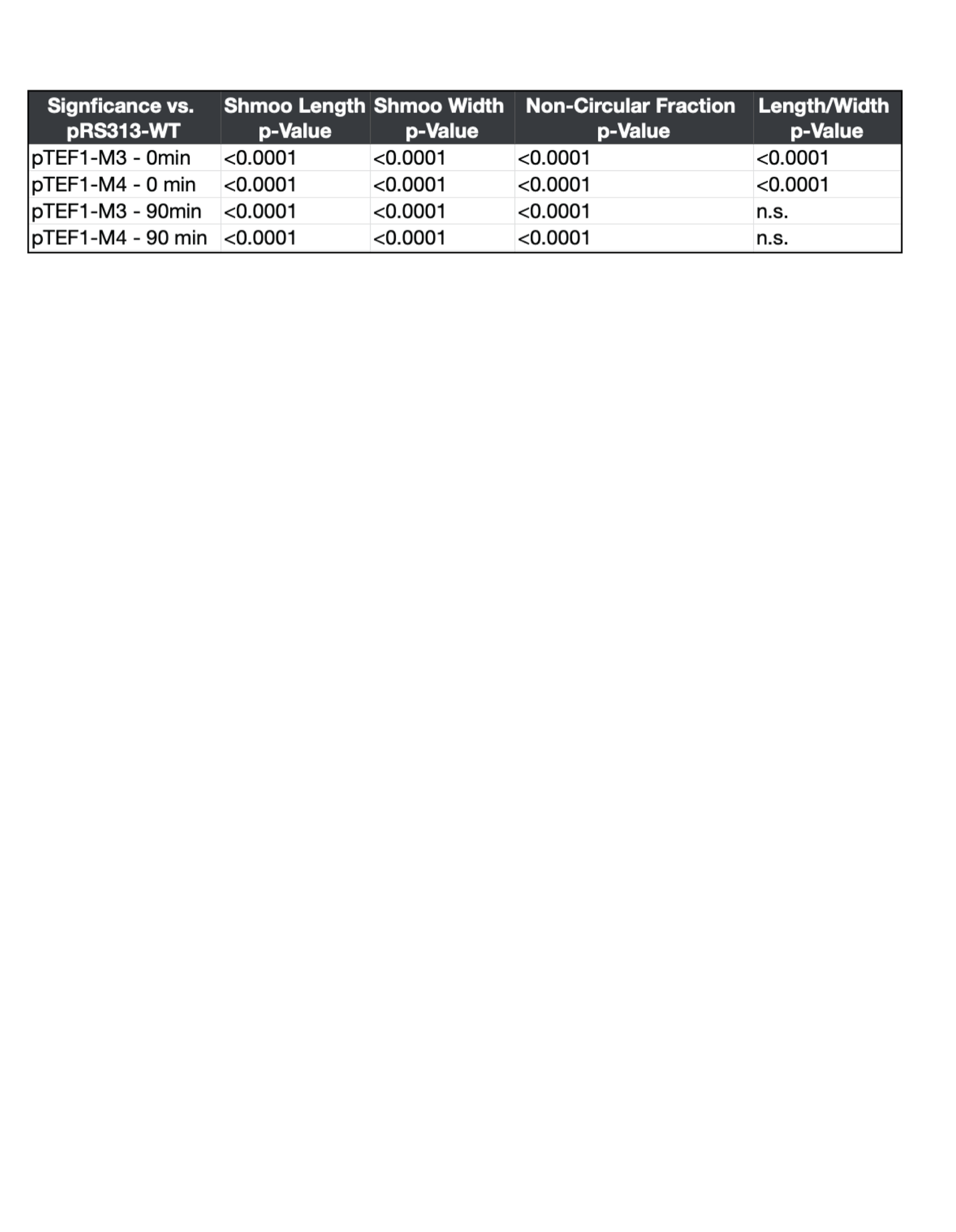
**Table S3.** **Table of helix-stabilizing and isometric control mutations incorporated into the HA-Ste18 N-terminal IDR.** In the sequence column, helix-stabilizing mutations are shown in red. C1 mutations intended to retain the intrinsically disordered state are shown in green. C2 mutations intended to provide an alternate helix-stabilizing mutation set are shown in orange. Predicted secondary structures are based on PEP2D predictions for the peptide in isolation (black, red, orange colors) or as an extension of the full-length Ste18 protein (where C = random coil and H = alpha helix, E= sheet). Residue positions that have been predicted as random coil in the peptide but predicted as alpha-helix in the full-length protein are shown in blue. Black dots track the point mutation positions in the peptide with respect to the secondary structure prediction. The mutation percentage relative to the peptide sequence length (Mut%) and the predicted alpha-helical percentage (Hel%) are shown to the right.


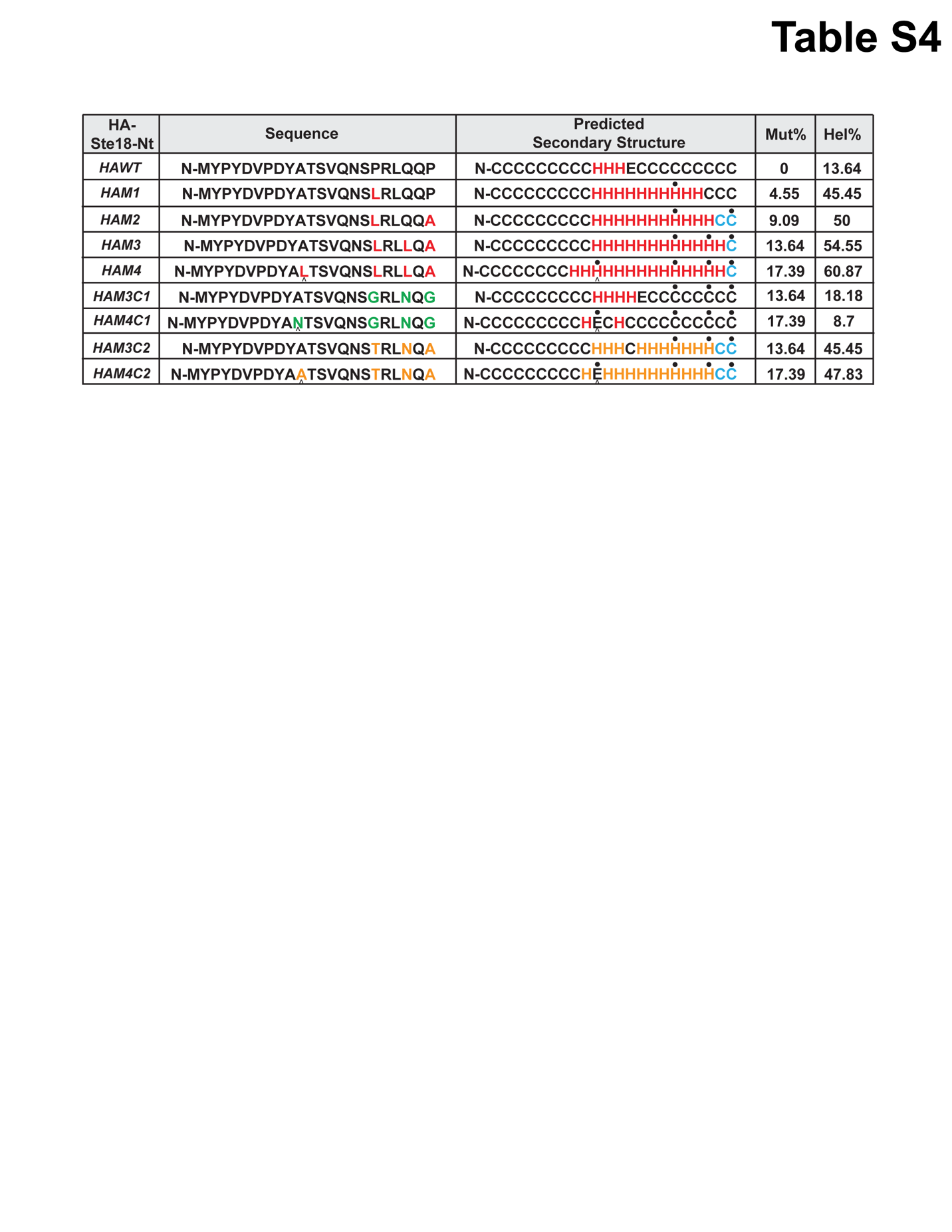


**Table S4.** **List of yeast strains used in this study.**

**
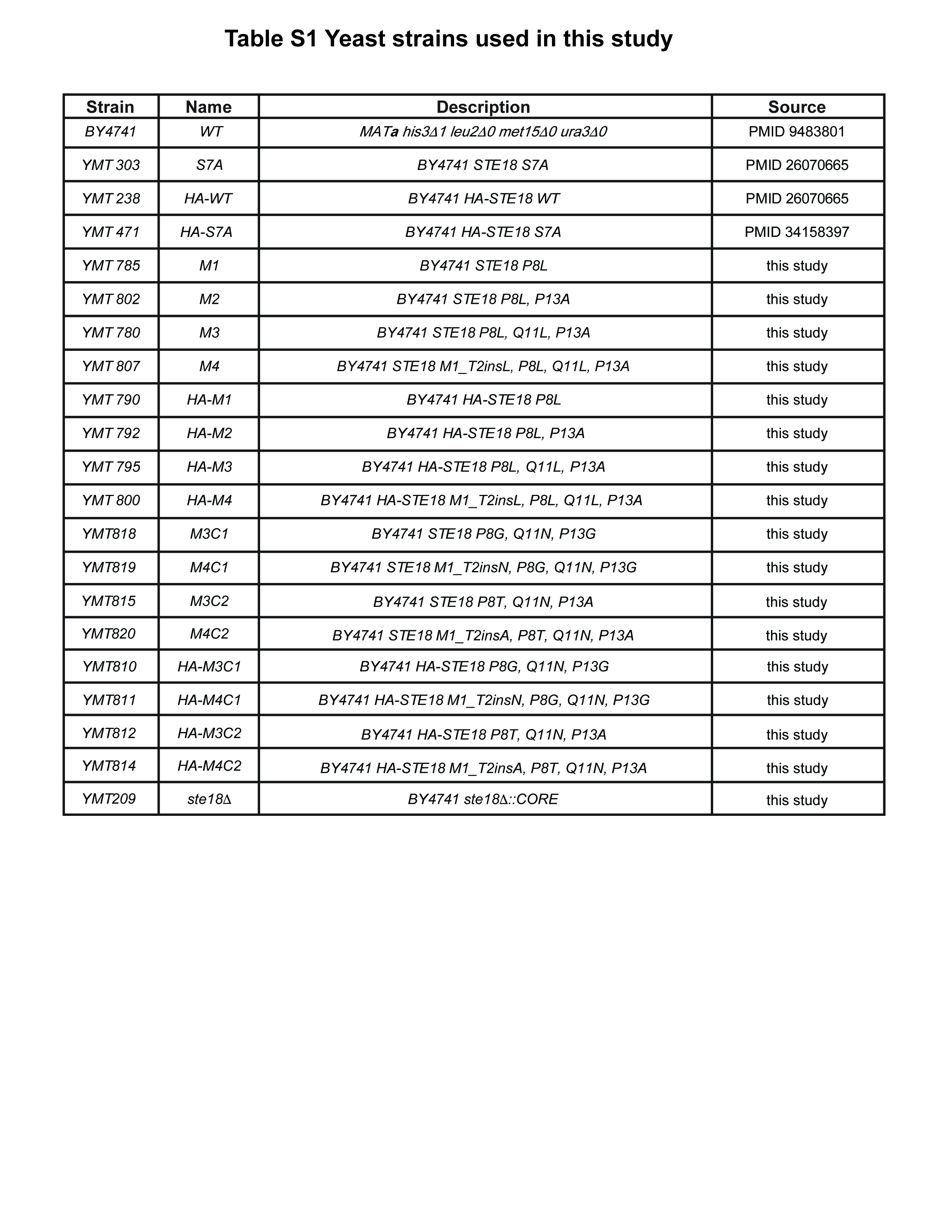
**


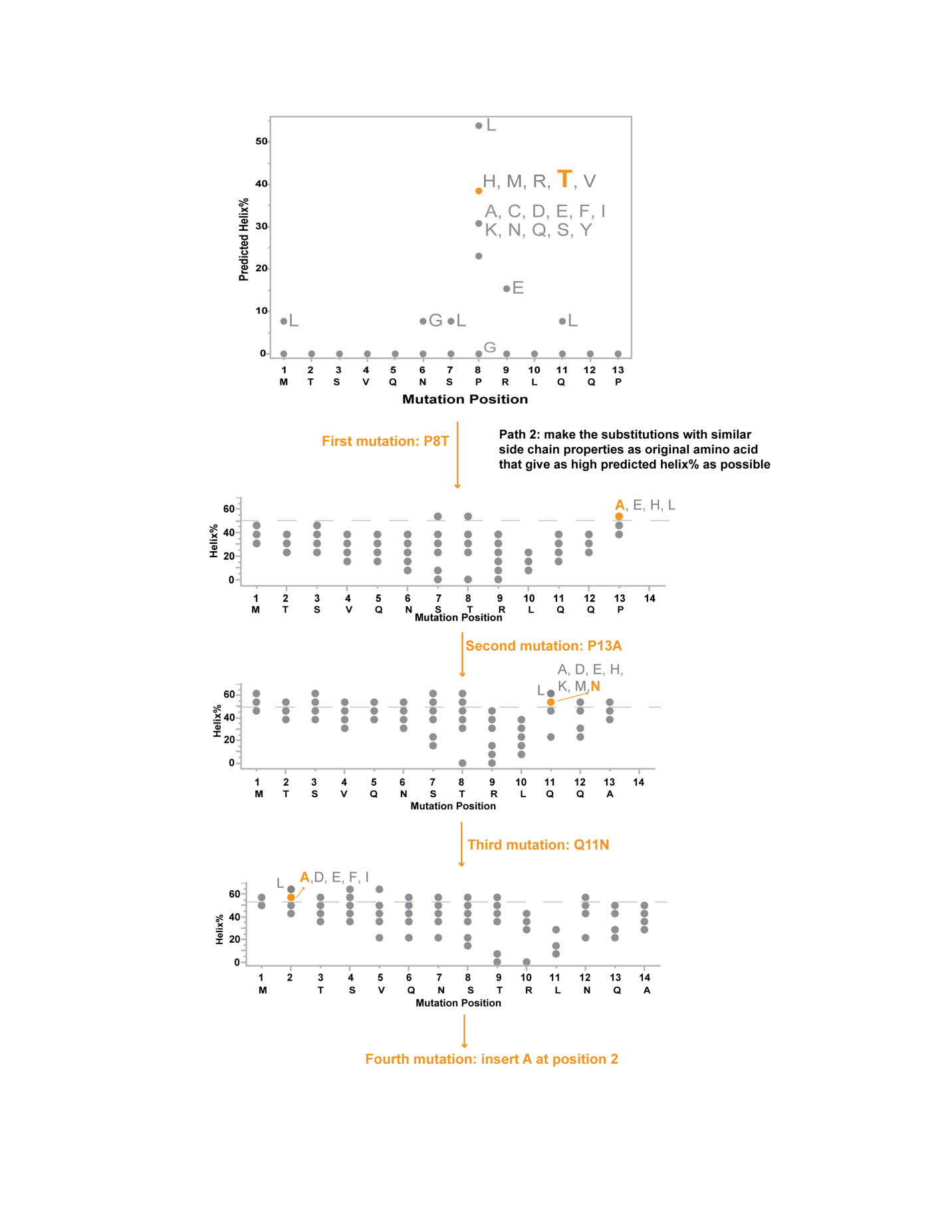


**Fig. S1.** **Path to introduce alternative helix-promoting substitutions to Ste18^Nt^.**


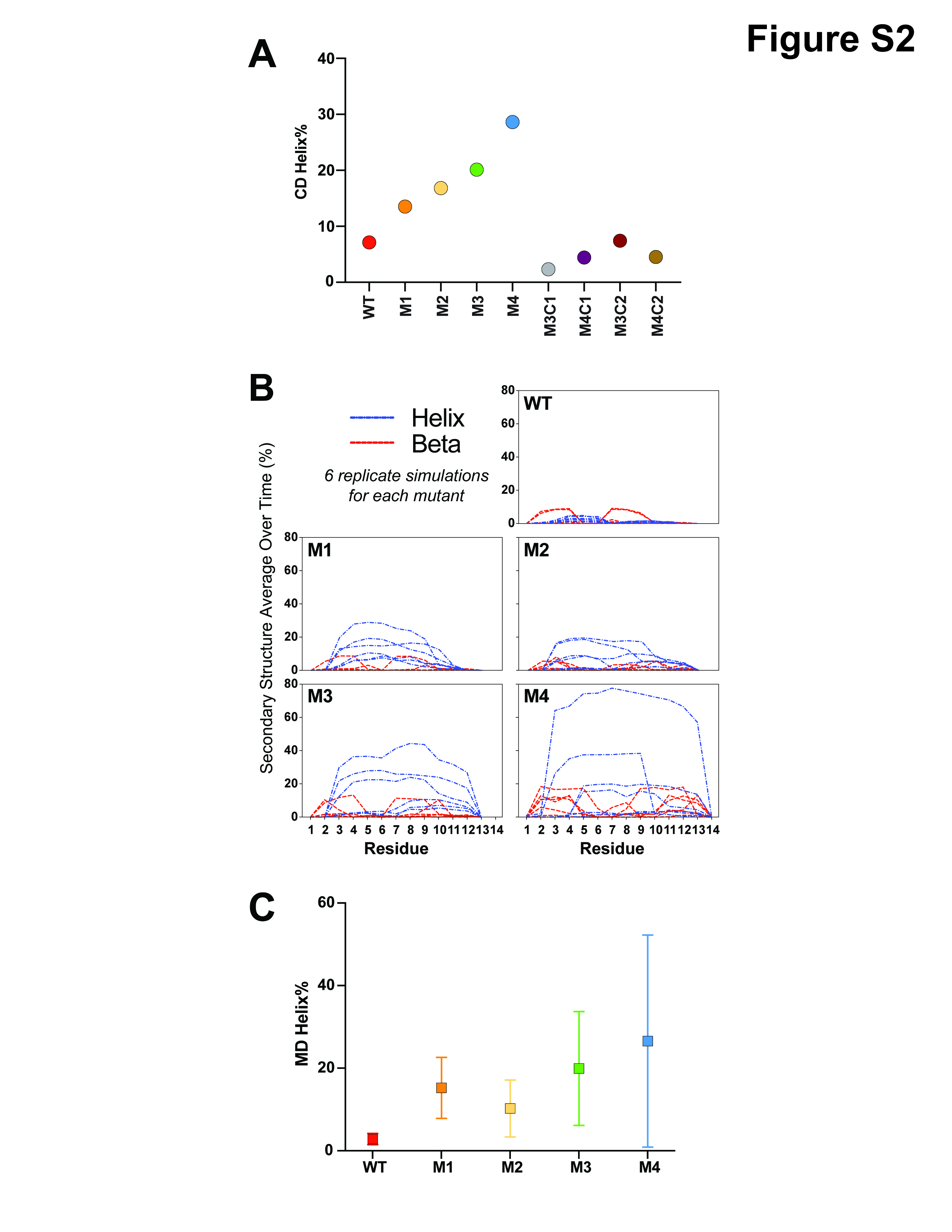


**Fig. S2.** **Molecular dynamics simulation replicates for WT and helix-stabilizing Ste18 mutant peptides M1-M4.** (A) Non-normalized quantification of peptide helix percentage from CD analysis (Fig. 3). (B) MD simulations for synthetic WT and helix-stabilizing M1-M4 peptides. Plots show the average secondary structure percentage (α-helix or β-hairpin) of 6 replicate simulations relative to the amino acid position in each peptide. (C) Non-normalized quantification of helix percentage in WT and helix-stabilizing peptides based on replicate MD analysis.


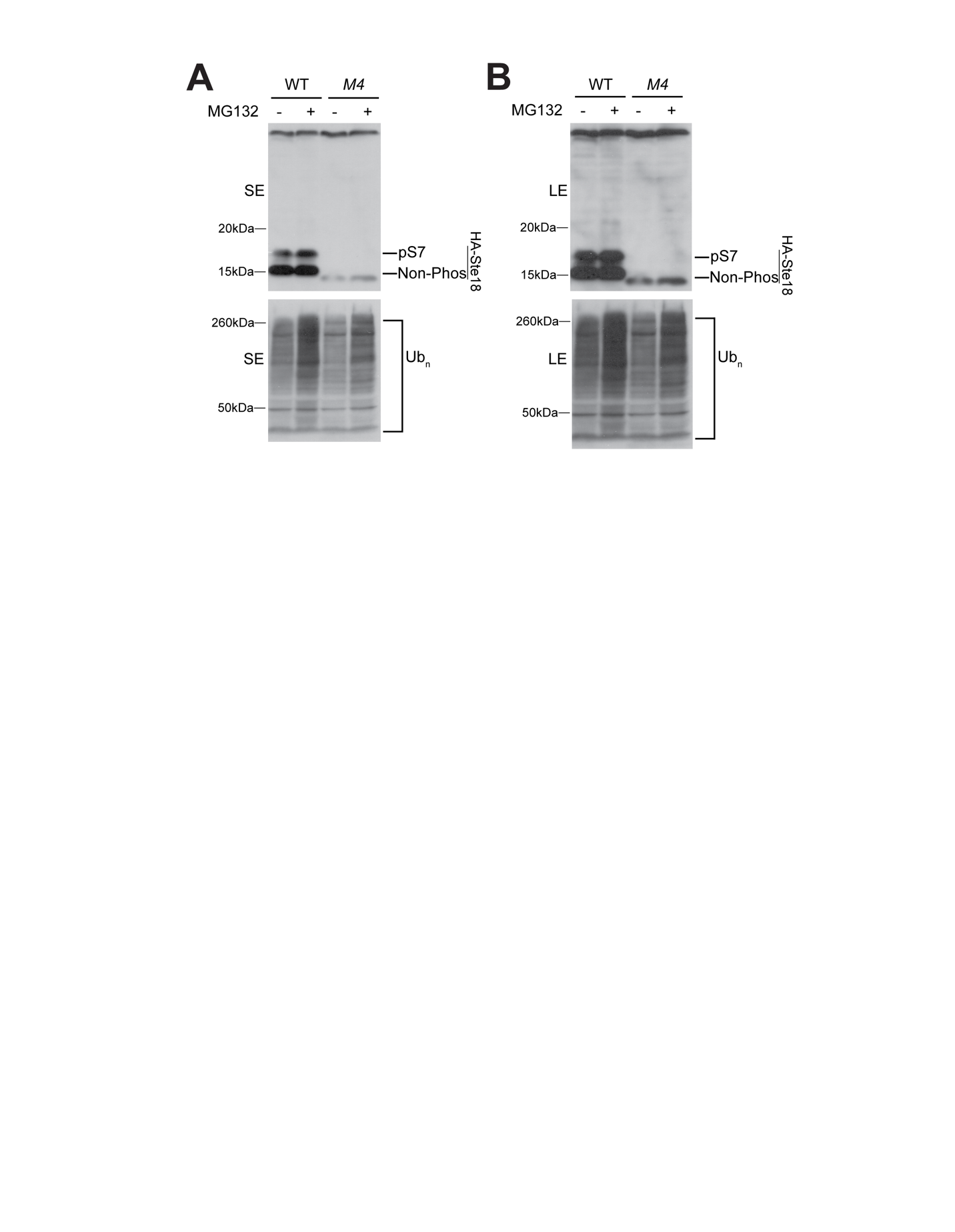


**Fig. S3.** **The effect of inhibiting the 26S proteasome on the cellular abundance of Ste18 mutant M4.** HA-Ste18^WT^ and HA-Ste18^M4^ cells were treated without or with 100μM proteasome inhibitor MG132 for 3.5 hours before subjected to SDS-PAGE and immunoblotting analysis using anti-HA and anti-ubiquitin antibody. Increase of ubiquitin after 3.5 hours of MG132 treatment indicated the proper function of MG132 as proteasome inhibitor. (A) Representative immunoblot of HA-Ste18^WT^ and HA-Ste18^M4^ cells without or with proteasome inhibition. (B) Representative immunoblot of long exposure in (A). pS7, Ste18 phosphorylation at Ser7; Non-Phos, non-phosphorylated Ste18; ubiquitin (Ub_n_). SE, short exposure. LE, long exposure.


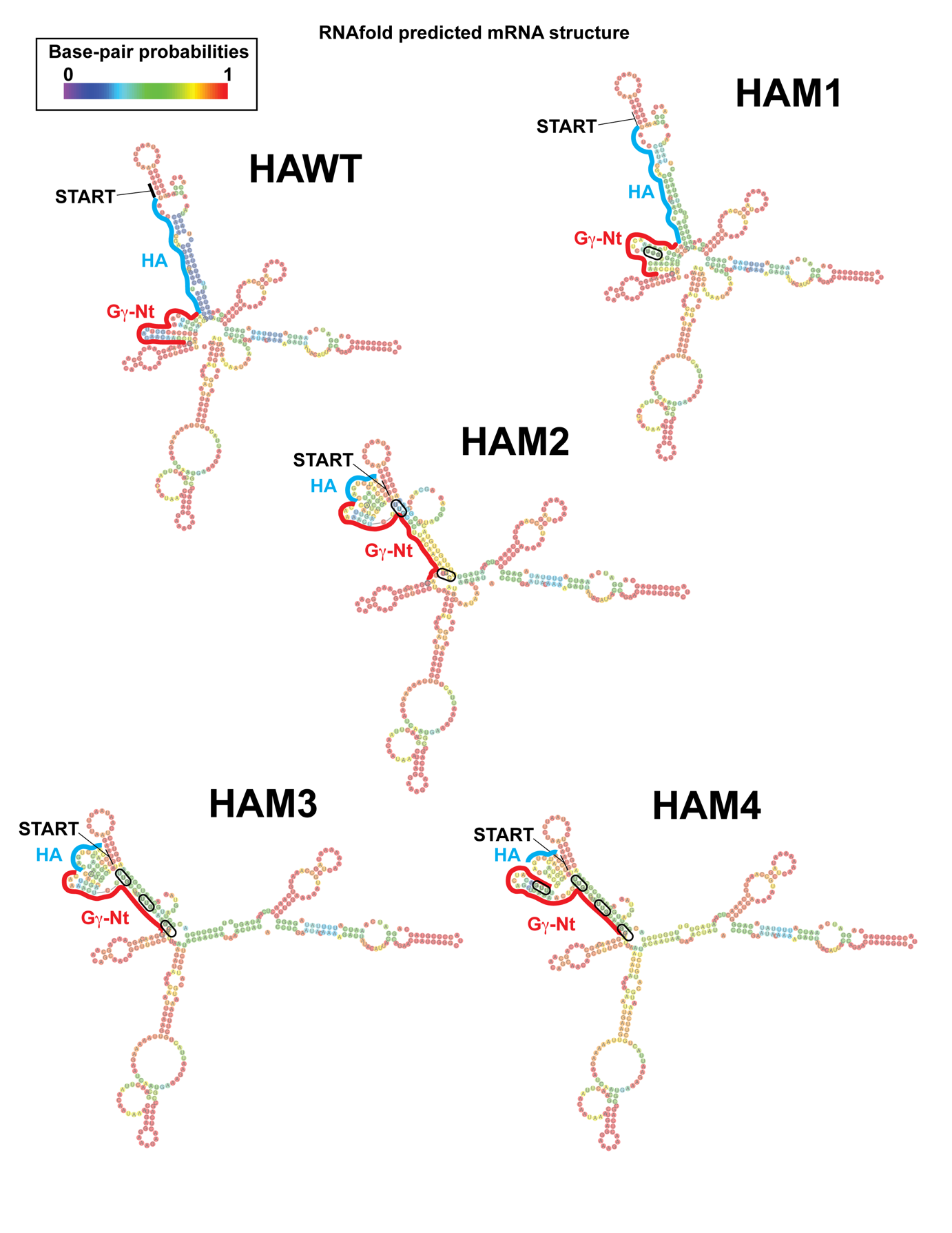


**Fig. S4.** **HA-tagged Ste18 wildtype and mutant isoforms mRNA secondary structure prediction.** mRNA secondary structures of HA-tagged Ste18 and mutant isoforms were predicted by RNAfold server using minimum free energy prediction. The structures were colored by base-pairing probabilities. For unpaired regions the color denotes the probability of being unpaired. START, start codon position; HA, HA tag position; Gγ-Nt, Ste18 N-terminal tail position.


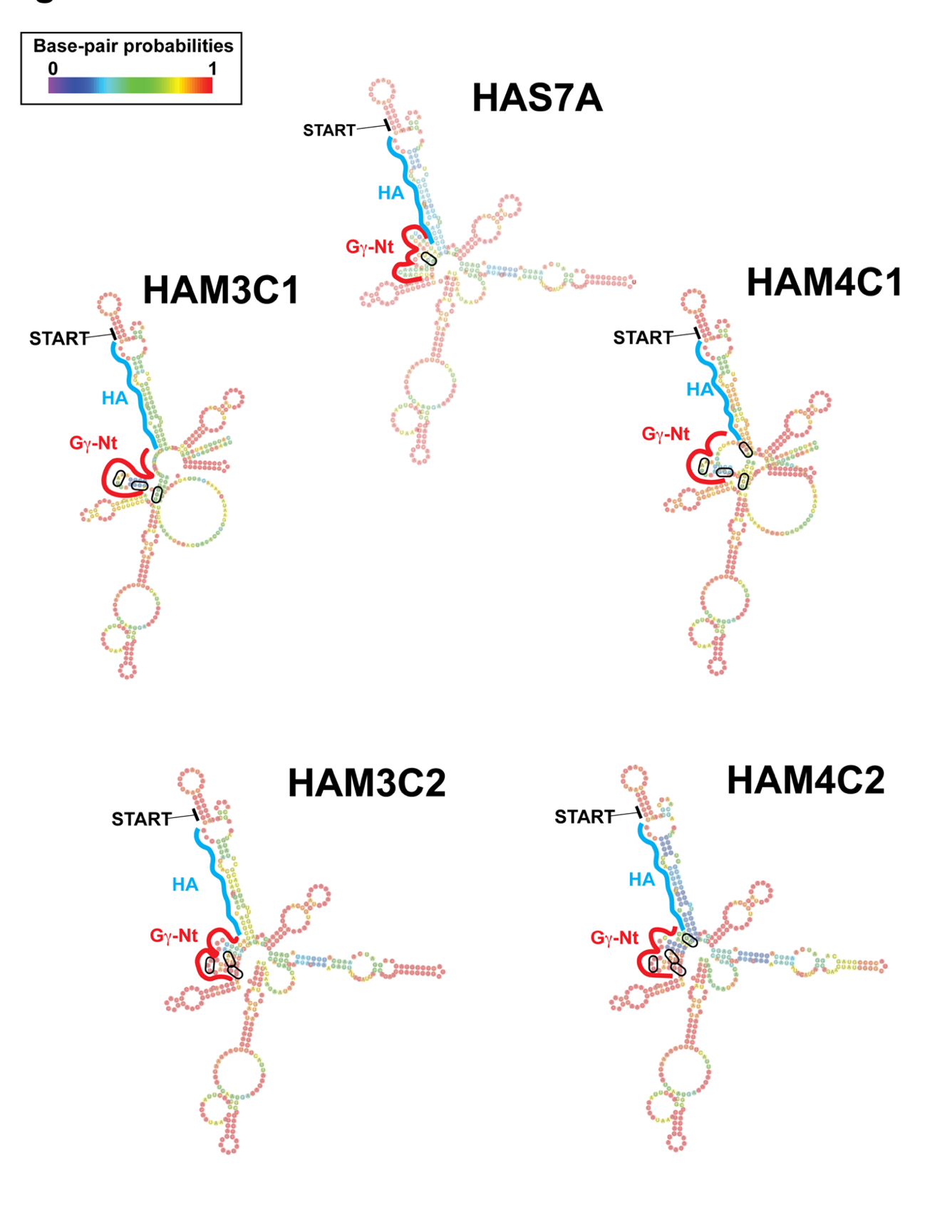


**Fig. S4.** **HA-tagged Ste18 wildtype and mutant isoforms mRNA secondary structure prediction.** mRNA secondary structures of HA-tagged Ste18 and mutant isoforms were predicted by RNAfold server using minimum free energy prediction. The structures were colored by base-pairing probabilities. For unpaired regions the color denotes the probability of being unpaired. START, start codon position; HA, HA tag position; Gγ-Nt, Ste18 N-terminal tail position.


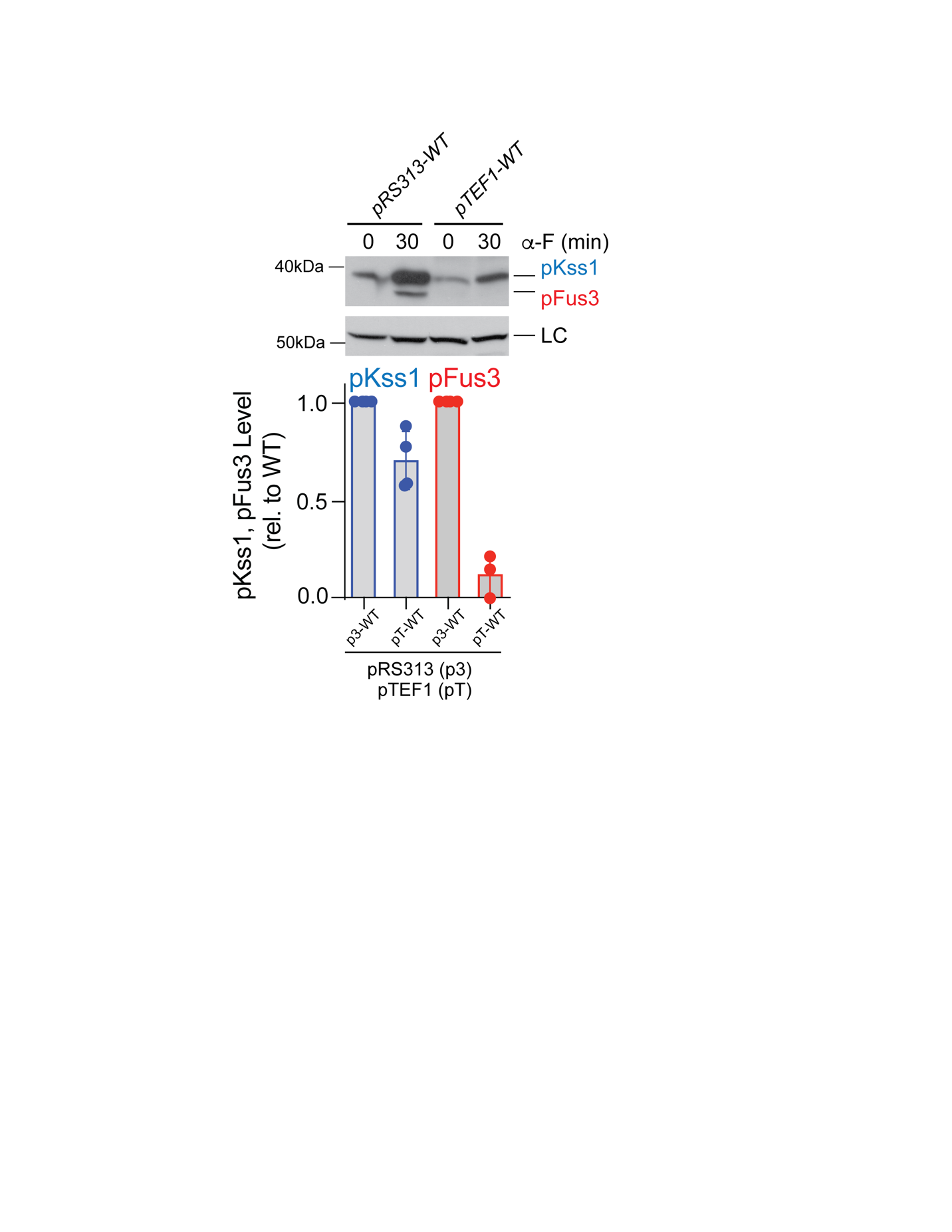


**Fig. S5. Immunoblot and image densitometry analysis for activated MAPK/Fus3 and MAPK/Kss1 in *pRS313-WT* versus *pTEF1-WT* cells 0 and 30 minutes post pheromone stimulation.** Representative phospho-MAPK immunoblot before and after 30 minutes treatment with 3μM α-factor and quantification of activated MAPKs level from experiments represented. *ste18Δ* cells transformed with *pRS313-STE18* or *pTEF1-STE18* were treated with 3μM α-factor followed by immunoblot analysis with anti-phospho-p44/42 MAPK antibody. Experiment was performed in 3 biological replicates. pKss1, phosphorylated Kss1. pFus3, phosphorylated Fus3. LC, loading control (yeast G6PDH). Blot shown for *pRS313-WT* is from the same gel/blot as shown in Fig. 6C and shown again here for comparison to *pTEF1-WT* that was on the same blot but not shown in Fig. 6C.

**
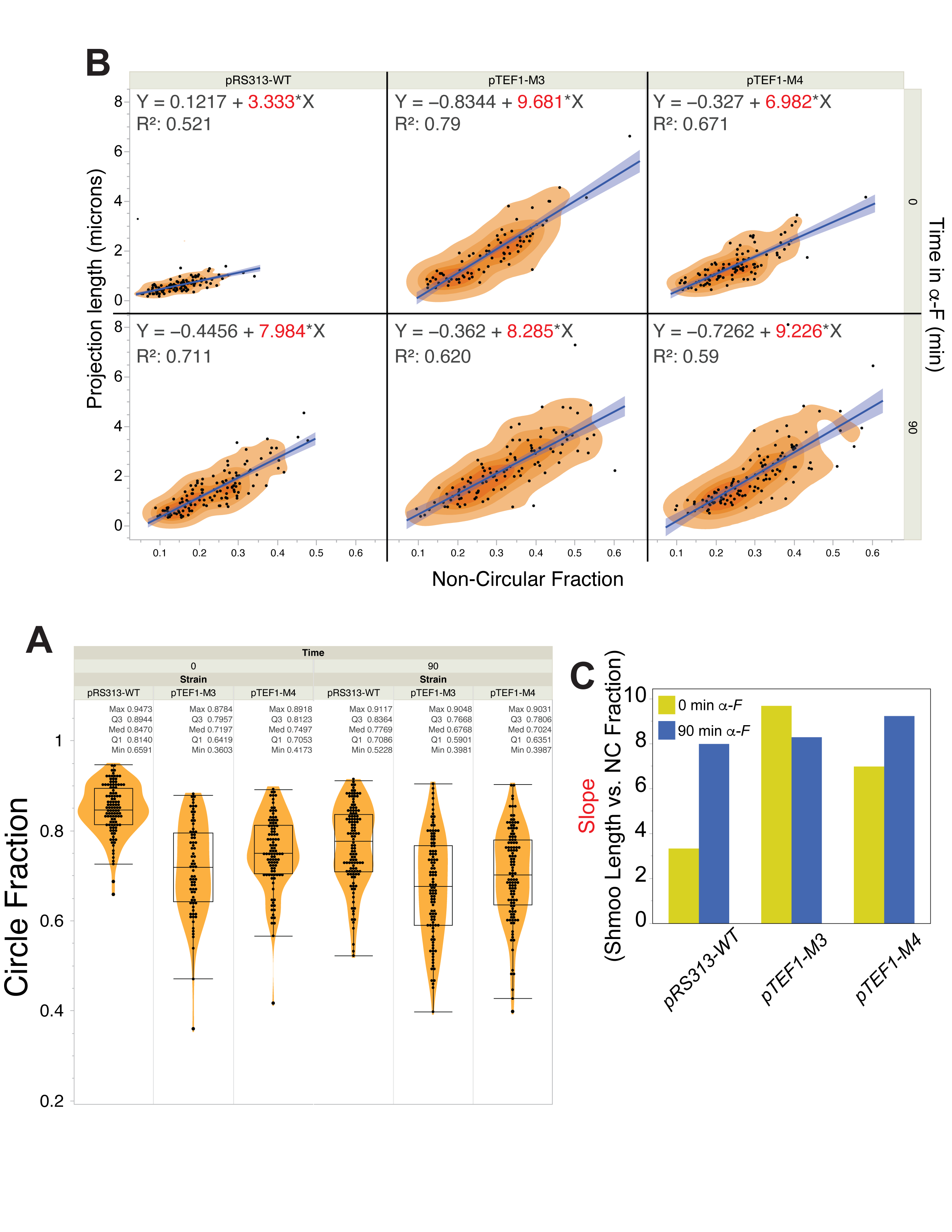
**

**Fig. S6.** **Comparison of circular fraction, non-circular fraction and projection length quantified from DIC microscope images across *pRS313-WT*, *pTEF1-M3* and *pTEF1-M4* yeast cells.** (A) Quantification of circle fraction without or with 90 minutes of pheromone stimulation across three cell types. Contour indicates data density. (B) Correlation analysis of non-circular fraction and projection length without or with 90 minutes of pheromone stimulation. Line of fit shows the linear regression with confidence intervals of 95%. Contour indicates data density. (C) Slope comparison of the fit line between *pRS313-WT*, *pTEF1-M3* and *pTEF1-M4* cells in B.


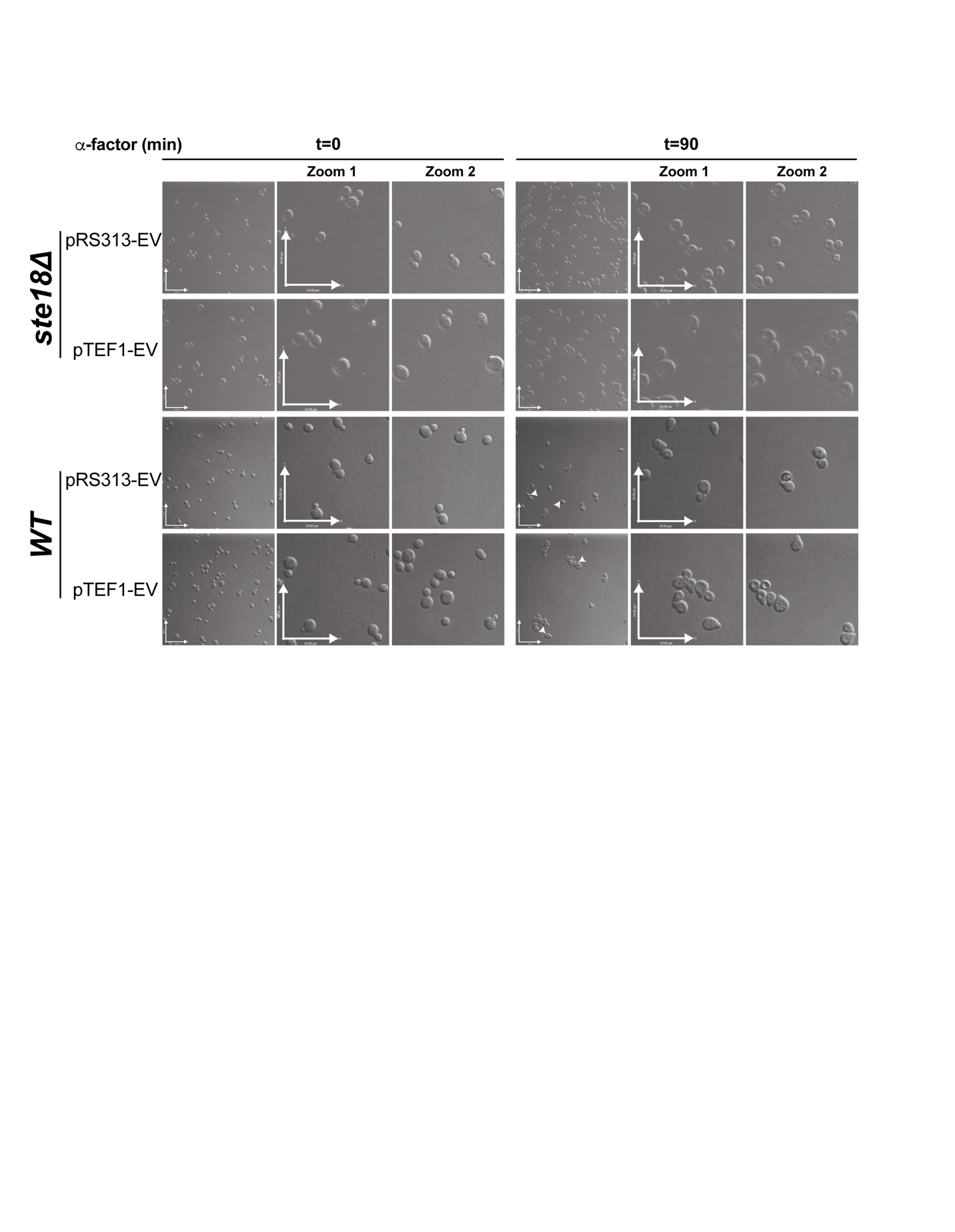


**Fig. S7.** **DIC microscope images of *ste18Δ* and WT BY4741 cells harboring *pRS313* or *pTEF1* empty vector treated with or without pheromone for 90 minutes.** Images are representatives of multiple images taken. Scale bars are 24μm x 24μm.

**
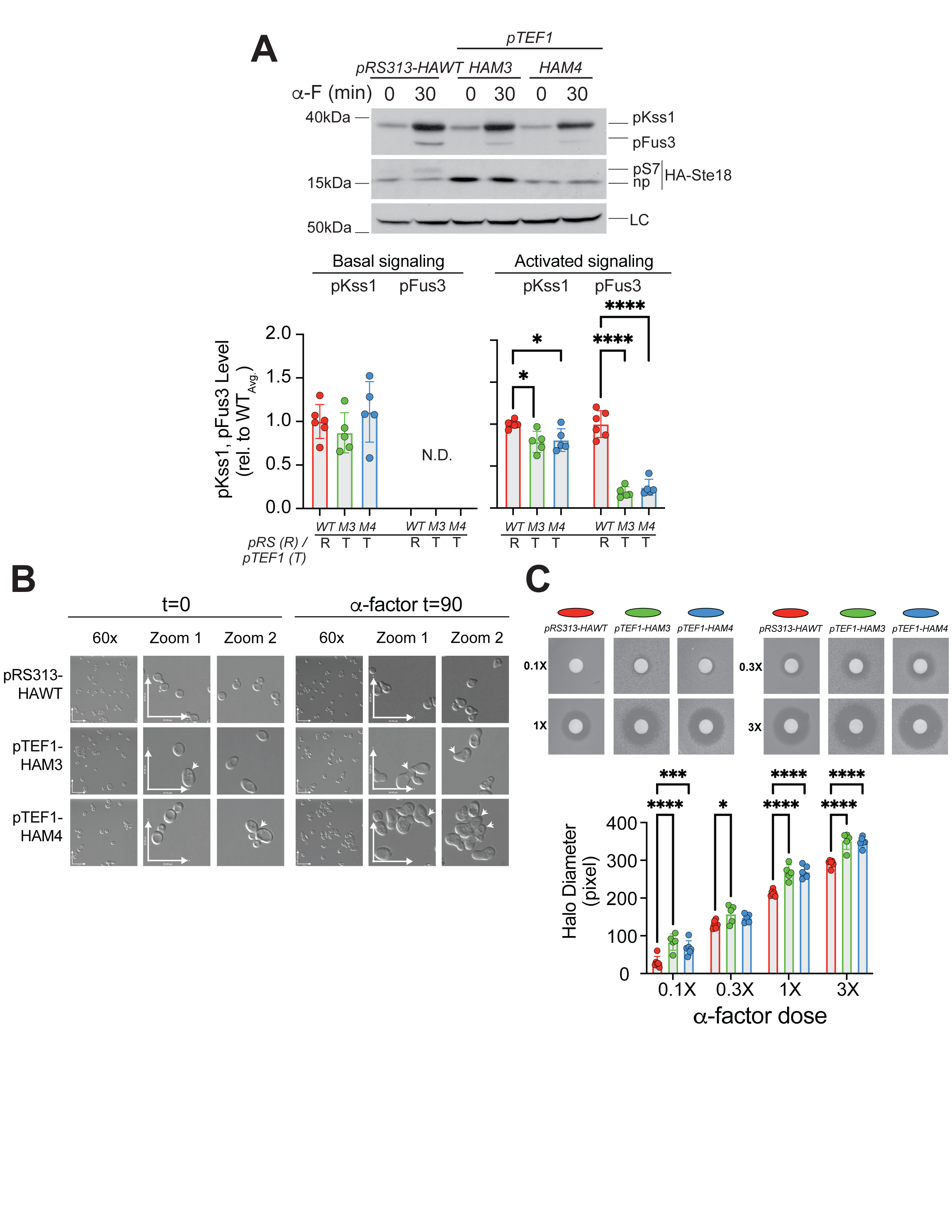
**

**Fig. S8. Effect of comparably-expressed HA-tagged Ste18-Nt helix-stabilizing mutants on pheromone-dependent MAPK activation, cell polarization, and cell cycle arrest.** (A) Representative immunoblot and quantification of phospho-MAPK before and after 30-minute treatment with 3μM α-factor. *ste18Δ* cells transformed with *pRS313-HASTE18* or *pTEF1-HASTE18-M3/M4* were treated with 3μM α-factor followed by immunoblot analysis with anti-phospho-p44/42 MAPK antibody and anti-HA antibody. The phospho-MAPK level of mutant was compared with the average phospho-MAPK level of wild-type running on the same blot. (B) DIC microscopy images of *ste18*Δ yeast harboring *pRS313-HAWT* or *pTEF1-HASTE18-M3*/*M4* expression plasmids before and after treatment with 3 μM α-factor for 1.5 hours. Images are representative of multiple images taken. Scale bars are 24μm x 24μm. (C) Representative image and quantification of halo assay for pheromone-induced cell cycle arrest. Sterile discs were saturated with 3X (3mM), 1X, 0.3X, or 0.1X α-factor. Experiments were repeated at least 3 times. Statistical significance was determined for MAPK and HALO results by two-way ANOVA and corrected for multiple comparisons using Tukey’s test. *P<0.0332, ***P<0.0002, ****P<0.0001. pS7, Ste18 phosphorylated at Ser7; np, non-phosphorylated Ste18. pKss1, activated Kss1; pFus3, activated Fus3. LC, loading control (yeast G6PDH).
